# DGCNN approach links metagenome-derived taxon and functional information providing insight into global soil organic carbon

**DOI:** 10.1038/s41522-024-00583-9

**Published:** 2024-10-26

**Authors:** Laura-Jayne Gardiner, Matthew Marshall, Katharina Reusch, Chris Dearden, Mark Birmingham, Anna Paola Carrieri, Edward O. Pyzer-Knapp, Ritesh Krishna, Andrew L. Neal

**Affiliations:** 1 IBM Research Europe, Sci-Tech Daresbury, The Hartree Centre, Warrington, UK; 2 STFC Daresbury Laboratory, The Hartree Centre, Warrington, UK; 3 Net Zero and Resilient Farming, Rothamsted Research, North Wyke, EX20 2SB UK

**Keywords:** Microbial genetics, Soil microbiology, Next-generation sequencing, Metagenomics

## Abstract

Metagenomics can provide insight into the microbial taxa present in a sample and, through gene identification, the functional potential of the community. However, taxonomic and functional information are typically considered separately in downstream analyses. We develop interpretable machine learning (ML) approaches for modelling metagenomic data, combining the biological representation of species with their associated genetically encoded functions within models. We apply our methods to investigate soil organic carbon (SOC) stocks. First, we combine a diverse global set of soil microbiome samples with environmental data, improving the predictive performance of classic ML and providing new insights into the role of soil microbiomes in global carbon cycling. Our network analysis of predictive taxa identified by classical ML models provides context for their ecological significance, extending the focus beyond just the most predictive taxa to ‘hidden’ features within the model that might be considered less predictive using standard methods for explainability. We next develop unique graph representations for individual microbiomes, linking microbial taxa to their associated functions directly, enabling predictions of SOC via deep graph convolutional neural networks (DGCNNs). Interpretation of the DGCNNs distinguished between the importance of functions of key individual species, providing genome sequence differences, e.g., gene loss/acquisition, that associate with SOC. These approaches identify several members of the Verrucomicrobiaceae family and a range of genetically encoded functions, e.g., related to carbohydrate metabolism, as important for SOC stocks and effective global SOC predictors. These relatively understudied but widespread organisms could play an important role in SOC dynamics globally.

## Introduction

Metagenomics has been proven to be an effective way to profile and explore highly diverse communities of microbes^1^. Metagenomics provides insight into the phylogenetic relationships between the taxa present in a sample and, through gene identification, can indicate the functional potential of the community. This allows us to test hypotheses regarding how microbial communities respond to environmental stimuli or change, e.g., climate and soil management, in the case of the soil microbiome. We know that community composition influences community function^2,3^, and these dynamics may be assessed by incorporating the taxonomic context of different functions (genes) into analyses. However, typically, taxonomic and genetic information are considered separately in analyses, although they are both derived from the same set of sequences.

The large volumes and high complexity of sequence data generated by metagenomics lend themselves to analysis using machine learning (ML), deep learning (DL) or artificial intelligence (AI) approaches^4^. Machine learning methods learn from input datasets, recognising and predicting patterns^5^. ML has been used for tasks (summarised in ref. ^6^) such as target or phenotype prediction (environmental or host-related, respectively)^7–12^, microbial feature classification (i.e., determining abundance, diversity, or distribution of microbiota)^13,14^, interaction analysis (e.g., to identify co-occurrence or microbe-metabolite interactions)^15,16^ and for identifying changes in microbiome composition^17^. Although ML has been applied extensively in recent years, its application to metagenomic datasets presents challenges. Data sparsity, the high degree of dimensionality and multiple correspondence of abundances between species make effective incorporation of derived features into models difficult. Generating explainable models to allow biological interpretation is also challenging. For target or phenotype prediction, it is imperative to understand not only which microbial species are associated or predictive, but also which potential functions they may be performing to have such effect. This requires the capability to link taxa to function directly for more informative biological interpretation. This has not been explored in previous works that focus on microbial taxa or functional capacity independently in models^18–20^. Furthermore, although explainable approaches are increasingly used, focus is typically on a few of the most important or predictive features, and little consideration is given to numerous less predictive features identified by a ML model.

Here, we focus on advancing the analysis of metagenomic data using interpretable ML methods. We test hypotheses using a soil metagenome dataset^19^ as an exemplar to study the role of soil microbes in the environment. Our hypotheses cover both technological innovation and the advancement of biological insight into associations between microbial communities and soil C stocks. The Bahram et al.^19^ dataset comprises whole shotgun metagenomes from 189 samples of topsoil collected from representative terrestrial regions and biomes across the world. This study correlated bacterial taxonomic diversity, composition, richness, and biomass with soil pH, nutrient concentration, and climate variables.

Soil is one of the world’s most important stores of organic carbon (C), with SOC stocks being greater than those of the atmosphere and terrestrial plants combined. C is released from soils to the atmosphere through biological respiration^21^ depending on factors such as soil structure, composition (e.g., porosity, water content etc.), management, vegetation, climate and nutritional inputs, which can regulate microbial activity^22^. This loss is largely offset by C incorporated into SOC through net primary production by plants. Given the magnitude of these stocks and fluxes, soil acts as an important regulator of climate^23^. Soil also supports most human food production, where evolving agricultural practices have been responsible for an estimated net loss of 75 Pg C from the top metre of soil^24^. Our use case for method development is therefore focused on this increasing need to understand SOC storage over a range of conditions to preserve or increase SOC storage.

Soil is increasingly understood as a biologically dynamic system^25^, where its function is determined by the interactions between its subsystems (such as mineral particles, organic matter, water, and microorganisms) and its surrounding systems. As such, we propose that the C content of soils can be understood more effectively if we combine climatic, edaphic and biological factors (species-level abundance and diversity, and functional potential). In doing so, we tackle issues around high-dimensional, interconnected feature sets for ML. We show the benefit of combining multiple codes for individual metagenomics bioinformatics processing steps and include complementary processing techniques such as alignment and classification in a single workflow. We use geospatial-temporal data extraction techniques via the IBM Environmental Intelligence Suite (EIS)^26,27^ (https://www.ibm.com/products/environmental-intelligence-suite/sustainability), formerly known as IBM PAIRS, to collect and exploit environmental data from a variety of sources to match our soil metagenome samples. Finally, we demonstrate that AI, in combination with graph-based methods, can effectively combine diverse features reflecting the biological complexity of the system into predictive models for edaphic parameters: here, we concentrate on C content estimation as an example. We show that the exploitation of explainable AI can provide biological insight into the most impactful features relating to C cycling and sequestration, facilitating the identification of predictive multi-feature fingerprints, including environmental factors, microbial species and, critically, their associated functions that might contribute to C cycling.

## Results

### Microbial phylogenetic diversity associates with habitat and geographical location

Bahram et al.^19^ demonstrated strong global-scale relationships between microbial taxonomic alpha-diversity, biomass, and several edaphic and climatic variables across eleven biomes. Prior to testing the ability of ML approaches to describe the relationship between microbial community function and SOC, we re-analysed the same 189 global soil metagenomic samples bioinformatically to confirm that we could detect these signatures, plus significant biome-specific differences in community phylogeny, which could be associated with SOC (Supplementary Note 1, Supplementary Tables 1–3, Supplementary Figs. 1–4 and Supplementary File 1). Weighted-*UniFrac* distances between bacterial, archaeal, and fungal communities in the independent samples were calculated based on the NCBI taxonomy for each species in the abundance matrix. Canonical correlation analysis (CCorA) in weighted-*UniFrac* space (see “Methods”; Supplementary Fig. 5) demonstrated significant phylogenetic distinctiveness between biomes for each kingdom (bacteria: trace statistic 2.18, *p* = 1·10^−^^5^; archaea: trace statistic 1.20, *p* = 1·10^−^^5^; fungi: trace statistic 2.64, *p* = 1·10^−^^5^). Canonical eigenvectors (*ν*) associated with edaphic and climatic factors are shown in Supplementary Fig. 5. For prokaryotes, phylogenetic differences between samples were most strongly associated with pH (*ν* = −0.785 and 0.773 for bacteria and archaea, respectively), but SOC also exerted an influence (*ν* = 0.726 for bacteria and *ν* = 0.716 for archaea). This is consistent with previous analyses for bacteria^19^. The phylogenetic distinctiveness of fungal communities in global soils revealed a different relationship. Fungi were most sensitive to SOC (*ν* = −0.588) and soil temperature (*ν* = 0.569), with pH exerting a reduced influence (*ν* = 0.535). The dominance of pH in determining phylogenetic beta-diversity that we observe is well established^28^.

### Feature preparation and normalisation for ML

Having established a clear association between SOC stocks and the phylogenetic distinctiveness of microbial communities in global soils, we proceeded to train and test a series of ML models to predict the average SOC stock for each geographical location (depth 0–5 cm), based upon separate taxon- and function-based abundance matrices (Supplementary Note 2; see “Methods”). Normalising by the number of mapped reads per sample ([counts per taxon or function/total number of reads mapped] * 1 × 10^6^) gave the best performance (Supplementary Fig. 6). We used this normalisation approach in all subsequent analyses and noted that functional and taxonomic models performed similarly. When we tested using alpha-diversity metrics to predict SOC, there was only a modest link between microbial alpha-diversity and global SOC (Supplementary Note 2; Supplementary Table 4; Supplementary Fig. 7).

Next, using a simple tabular concatentation, we combined taxonomic and functional feature abundances with alpha-diversity feature sets (191,993 features) and trained and tested a series of ML models to predict SOC content (see “Methods”). The best model (selection described in Supplementary Note 2) was generated using a Random Forest (MAE of 30.3 after CV, 18.4 on training and 23.9 on the test dataset, Supplementary Fig. 8a). The model showed good agreement between measured and predicted SOC levels for the test dataset (*r* = 0.81; Supplementary Fig. 8b). Performance was similar when either taxonomic or functional features were used. The CV MAE equates to an ~8% error since the range of SOC measurements across our samples was 8–392 g kg^−1^.

### Environmental data profiling for soil microbiome collection

**Fig. 1:**
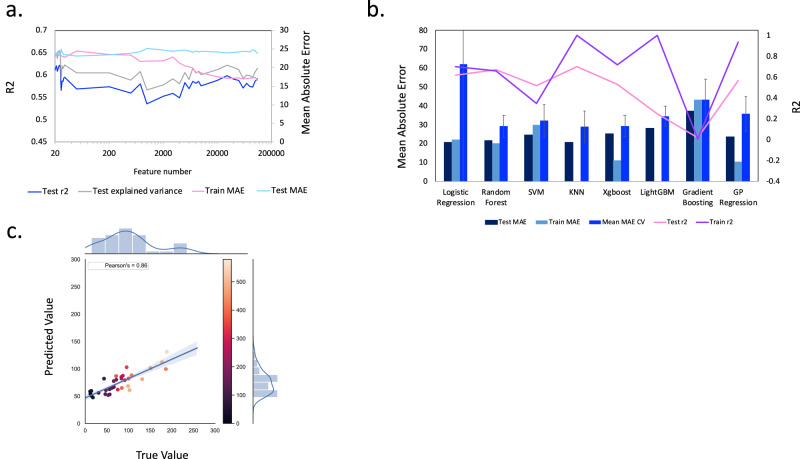
Comparing ML models for combined environmental, taxonomic and functional abundances plus alpha diversity metrics—22 features after feature selection. **a** Mean Absolute Error (MAE) on training and held-out test data plus the *r*^*2*^ and explained variance on the held-out test data using the “best” performing Random Forest model for each reduced feature subset size as feature number is reduced on the basis of univariate linear regression tests. **b** ML model performance for a range of regressors and metrics, as described in Supplementary Fig. 6 (see legend at bottom of figure). ML models were trained using the combined features for environment, normalised taxonomic abundance, normalised functional abundance and alpha diversity metrics after feature selection shown in (**a**) down to the 22 most predictive features to predict SOC levels. Supplementary Table 6 numerically summarises key values from the same data shown in the plot. **c** For the same 22 features, this plot shows the true versus predicted values for SOC content (g kg^−1^) for each of the soil metagenomic samples from the held-out test dataset. This plot refers to the best-performing model defined from (**b**), i.e., SVM. The Pearson correlation coefficient (*r*) is also shown (0.86) (*p* = 2.9·10^−1^^1^), using a Kendall rank correlation test the coefficient was 0.64 (*p* = 5.9·10^−^^8^). The colour bar shows the interquartile range for the predicted soil organic carbon contents—of which we predict the mean value—at each location, this depicts the confidence we have in the target variable.

The environmental variables that we selected for ML included (Supplementary Note 3; Supplementary Fig. 9; Supplementary Table 5): soil clay, soil sand, soil silt, soil pH, soil class, soil temperature (0–5 cm depth), soil water (0–7 cm), mean annual air temperature, total annual rainfall, year/month of sampling, difference in total rainfall and temperature (between 20-year average and day of sampling), and habitat of sample. We combined these variables with microbial function and species abundance counts. Using this feature set, the best ML model gave highly comparative performance to previous models, and we noted there was still overfitting of our model (Supplementary Note 3). In fact, many of the ML algorithms produced overfitted models throughout our investigations (e.g., in Supplementary Figs. 6 and 8a, we rarely see close agreement between the training, test and CV MAE). Consequently, we used feature selection to identify a subset of the most predictive features (Supplementary Note 3; Fig. 1a, see “Methods”). Twenty-two features were identified, including two environmental features. We re-trained, tested and tuned a range of ML regressors with this reduced feature set. Fig. 1b (Supplementary Table 6) shows that this generally reduced model overfitting. Prioritising minimisation of overfitting, i.e., closest congruence between test, train and CV MAE, alongside the lowest MAE on CV, our best model is produced with Support Vector Machine (SVM). The SVM model yielded a MAE of 32.1 on CV (SD 8.8), 29.9 on the training data and 24.6 on the test data. It also showed a higher correlation between measured and predicted SOC levels (*r* = 0.86; Fig. 1c).

**Fig. 2:**
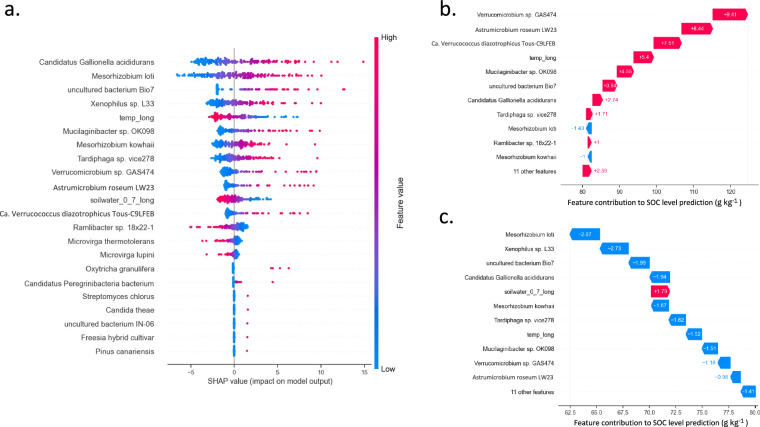
Model explanation for the ML prediction of soil organic carbon levels using twenty-two features. Using SHAP to interpret our best ML model generated with SVM. **a** summarises the ranking of the most impactful features for prediction of SOC level for each metagenomic sample’s geo-location, considering all samples in the dataset (training and test). This is known as a general explanation of the model for predicting SOC level across all of the samples (global explanation). Feature value equates to the normalised read count or abundance per feature for microbial taxon/function. For “*temp_long*” the feature value equates to the 20-year average air temperature (°C) and for “*soilwater_0_7_long*” the 20-year average soil water content (m^3^ m^−^^3^). When the abundance per taxon/function is high (red) and it has a positive SHAP value, this high frequency is driving the association with a high SOC content. This is often coupled with the situation where the lower frequency of the same taxon/function per sample has a negative SHAP value, so the absence of the species or function is driving the prediction of a lower SOC level. On the contrary, when the frequency of the feature per sample is high (red) and has a negative SHAP value, the high frequency or abundance is driving the prediction of a lower SOC level. This is often coupled with the situation where the lower frequency of the feature (blue) has a positive SHAP value, so the absence of the species/function is driving the prediction of a lower SOC level. **b** Here we use a waterfall plot to focus on the explanation for the prediction of a specific sample S234 from the Hanhijängän-Pierkivaaranjängän peatland protection area, Finland, (habitat: Boreal Forest, location: latitude 69.17°/longitude 27.00°) that the model predicted a soil organic carbon level of 124 g kg^−1^ for when its measured value is 194 g kg^−1^ (local explanation). This plot shows the top 12 most influential features that contributed to the SOC level prediction by the model for this sample. The bottom of a waterfall plot starts as the expected value of the model output (80 g kg^−1^), and then each row shows how the positive (red) or negative (blue) contribution of each feature moves the value from the expected model output over the background dataset to the model output for this prediction in g kg^−1^ of SOC. **c** This plot is the same as (**b**) however for a different sample S72 from the San Felasco Hammock Preserve State Park, Florida, US (habitat: temperate deciduous forests, location: latitude 29.74° N/longitude −82.44°) that the model predicted a SOC level of 62 g kg^−1^ for when its measured value is 49 g kg^−1^ (local explanation).

Feature selection provided the first insight into the organisms and environmental factors that were important for predicting SOC. But, on their own, they provide no information regarding their contribution to the final SVM model predictions and thus their likely importance for SOC stocks. To generate these explanations, we applied Shapley Additive exPlanations (SHAP) to infer the contribution of each of the twenty taxa and two physical features to the best predictive SVM model (Fig. 2a)^29^. Although the twenty-two features all correlated with SOC level, the five features associated with the smallest SHAP impact values (bottom five lines in Fig. 2a) are low abundance taxa, appear in fewer samples and have a comparatively low impact on the predicted SOC level by the ML model at a global level, perhaps because these features were not detected in the majority of soil metagenomes. Table 1 details the remaining seventeen features that were most impactful for the ML model. It highlights that the prioritisation of features by the SVM model (ranking by their global SHAP impact values) differs from the ranking by direct correlation with SOC.

**Table 1:** Top identified most predictive features

Rank (regression)	Rank (SHAP- ML)	*r* for SOC content	Species	Relevance
1	1	0.624	*Candidatus* Gallionella acididurans	A β-proteobacterium. Previous analysis of this species found that genes required for autotrophic carbon fixation via the Calvin cycle were present, while no pathway for nitrogen fixation was revealed. An aerobic chemolithoautotroph of a group of psychrotolerant iron- and sulfur-oxidising acidophiles^78^
2	8	0.572	*Tardiphaga* sp. vice278	Nitrogen-fixing α-proteobacterium. The genome of this species contains footprints of horizontal operon transfer (HOT) of complete gene clusters encoding BChl- and xanthorhodopsin (XR)-based dual phototrophy. It has both a photosynthesis gene cluster and an XR operon and contains RuBisCO genes, a phosphoribulokinase gene, and a soluble methane monooxygenase gene, pointing to the metabolic potentials for carbon fixation via photoautotrophy and methanotrophy^79^
3	4	0.504	*Xenophilus* sp. L33	A β-proteobacterium from the genus *Xenophilus* that decreases abundance at elevated CO_2_^80^. An altered soil microbial community at elevated CO_2_ leads to loss of soil carbon, so we could link *Xenophilus* decrease to lower soil carbon levels^81^.
4	5	−0.499	Temp_long	20-year average air temperature for a sampling site
5	7	0.495	*Mesorhizobium kowhaii*	Nitrogen-fixing α-proteobacterium commonly isolated from root nodules of leguminous plants^30,44,45^
6	2	0.492	*Mesorhizobium loti*	Nitrogen-fixing α-proteobacterium commonly isolated from root nodules of leguminous plants^30,44,45^
7	16	0.465	*Oxytricha granulifera*	A Spirotrich ciliate protozoan. Abundance was positively correlated with soil moisture, salinity, organic matter, total nitrogen, total phosphorus, and sulfate, but negatively with pH and total potassium. Soil ciliates have participated in the decomposition of benthic residual deposit and the formation and development of mangrove soil and accelerated the mineralisation processes of carbon, nitrogen, and other mineral nutrient elements^82^.
8	9	0.456	*Verrucomicrobium* sp. GAS474	*S*low-growing member of the order Verrucomicrobiales originally isolated from forest soil of a long-term soil warming experiment in Massachusetts, US^46^, which is closely related to *Astrumicrobium roseum* LW23^39^. Verrucomicrobia are highly relevant to SOC levels since evidence exists that they play critical roles in environmental carbon cycling and (poly)saccharide degradation, as they can have high coding densities for glycoside hydrolase genes^39^.
9	10	0.452	*Astrumicrobium roseum* LW23	Formerly referred to as Verrucomicrobia bacterium LW23. See information relating to *Verrucomicrobium* sp. GAS474, above
10	3	0.439	uncultured bacterium Bio7	Genome sequence investigation revealed two associated biological process-related GO terms for this species, the majority sub-class (for 50% of the terms) was related to metabolic processes, of which some of the main sub-terms related to organic substances (25%) and nitrogen compounds (25%).
11	12	0.433	*Candidatus* Verrucococcus diazotrophicus Tous-C9LFEB	*Candidatus* Verrucococcus diazotrophicus Tous-C9LFEB, formerly referred to as Verrucomicrobia bacterium Tous C9LFEB. See information relating to *Verrucomicrobium* sp. GAS474, above. Genome is asociated with 218 glycosyl hydrolases, nitrogen fixation and efficient degradation of recalcitrant compounds such as chitin, cellulose, xylan, starch, or mannan^49^
12	6	0.428	*Mucilaginibacter* sp. OK098	The genus *Mucilaginibacter* belongs to the family Sphingobacteriaceae, which are nonmotile, rod-shaped, and exopolysaccharide-producing (EPS) bacteria. The matrix produced by EPS around microbial cells has the capability of shielding them against antimicrobial compounds and heavy metals; EPS matrix can also retain water, protecting microbes and the environment against drought. In addition, other functions, such as adhesion, communication with other microbes and plants, antioxidant, aggregation, carbon storage, and entrapment of nutrients have also been reported^83^
13	14	−0.404	*Microvirga thermotolerans*	Thermo-tolerant α-proteobacterium *Microvirga thermotolerans*, originally isolated from rice paddy soil in northwest China^31^. This species has a growth optimum of 40 °C, i.e., prefers warmer climates^31^, Since we know that C storage declines strongly with mean annual temperature increase^84^, this species abundance correlates negatively with predicted carbon level for the soil.
14	15	−0.378	*Microvirga lupini*	Nitrogen-fixing α-proteobacterium *Microvirga lupini* originally isolated from nodules of *Lupinus texensis* roots in Texas, US^30^
15	13	−0.374	*Ramlibacter humi* strain 18x22-1	β-proteobacterium, aerobic and nonmotile, originally isolated from a tropical forest soil in Hainan Province, China^32^. After addition of biological fertiliser to soil *Ramlibacter* levels were seen to decrease^85^.
16	11	−0.053	Soil-water-0-7cm_long	20-year average of soil water content at sampling site at a depth of 0–7 cm
17	17	0.029	*Candidatus* Peregrinibacteria bacterium CG_4_10_14_0_2_um_filter_38_24	Recent work expanded the Peregrinibacteria (PER) II/III RubisCO diversity. Detection of form II/III RubisCO and nucleoside metabolism gene transcripts from a PER supports the operation of this pathway in situ. We demonstrate that the PER form II/III RubisCO is catalytically active, fixing CO_2_ to physiologically complement phototrophic growth in a bacterial photoautotrophic^86^.

Features identified using *f_regression* univariate linear regression tests with regard to the target variable for prediction, SOC levels (g kg^−1^). Features are first ordered by *f_regression* (scikit learn) defined correlation with the target variable and second by their associated impact values on the best performing SVM model as calculated by SHAP. The five features that did not contribute strongly to the SVM model are not included in the table. The Pearson correlation coefficient (*r*) is shown for each feature (species abundance) versus the target variable SOC content (as calculated by the scikit learn package *r_regression*); all features have associated *p*-values for these correlation coefficients of less than 0.001 except for Soil-water-0-7cm_long and *Candidatus* Peregrinibacteria bacterium. Four of the identified bacterial taxa are associated with nitrogen fixation, three are from the order Verrucomicrobiales.

Seventeen of the twenty taxon-derived features in Fig. 2a show positive associations, indicating that greater abundance of these species in a sample is associated with higher SOC predictions. Only the Hyphomicrobiales *Microvirga lupini*^30^ and *Microvirga thermotolerans*^31^, and the β-proteobacterium *Ramlibacter humi*^32^ deviate from this trend. For these organisms, greater abundance is associated with lower predicted SOC. Several Hyphomicrobiales were predictive and had both positive and negative associations with SOC content, highlighting the importance of species-level investigation of SOC. Abundances of *Microvirga thermotolerans* and *Microvirga lupini* across the samples were negatively correlated with those of the other Hyphomicrobiales *Tardiphaga* sp. vice278, *Mesorhizobium kowhaii* and *Mesorhizobium loti* (Supplementary Fig. 10a–f, r values from −0.28 to −0.44, *p* < 0.0001).

Two of the top eleven most predictive features are related to environmental factors, i.e., long-term averages of temperature and soil water content, highlighting the impact of long-term environment on SOC (Supplementary Note 4). The 20-year average air temperature for a locale was a more effective predictor of SOC content than the temperature on day of sampling. This is in line with longer timelines (years-decades) for the turnover of the majority of SOC pools^33^. Three of the top features in Table 1 were species from the Verrucomicrobiaceae family (*Verrucomicrobium* sp. GAS474, *Astrumicrobium roseum* LW23 and *Ca*. Verrucococcus diazotrophicus Tous-C9LFEB). All were significantly positively correlated with SOC content (Supplementary Fig. 11a–c, *r* ranging from 0.43 to 0.46, *p* < 0.0001). Supplementary Fig. 11d, e shows different patterns of Verrucomicrobiaceae relative abundances between the three species that discriminate high and low SOC content with statistical confidence; this might be why all three species were retained for predictions.

For individual samples (geographical locations) local SHAP explanations were used to compare features contributing to their specific SOC predictions. To demonstrate, we compared the features contributing to SOC predictions for two samples having higher (194 g kg^−1^) and lower (49 g kg^−1^) SOC, with respect to the range across the samples. Figure 2b, c shows that nine of the eleven most influential features for SOC prediction are shared across the two samples, and all features that were important to the samples individually were also globally important across all samples, i.e., observed in the top fifteen of Fig. 2a. Typically features show a mirror image in terms of feature values or abundances, i.e., a feature driving a higher predicted SOC in one sample is coupled to the same feature driving a lower SOC in the other sample based on contrasting (high/low) levels.

CCorA indicated that fungal communities were particularly sensitive to SOC stocks. There are large differences in the number of fungal taxa in soils (maximum ^0^*D*_fungi_ = 2000) compared to prokaryotes (maximum ^0^*D*_bacteria_ = 40,000). Nonetheless, we expected to identify fungi in the most predictive features for SOC given that ML methods should be unaffected by taxon number, prioritising predictivity. One fungus, the yeast *Candida theae*, was in the most predictive features (Fig. 2a). However, low sequencing depths (3.5 million read pairs per sample on average) are likely to result in under-representation of fungal communities which would diminish the predictivity of these taxa, even if they are biologically relevant. Deeper sequencing is required to test this notion. Previous studies of the same dataset we are using primarily linked fungal biomass and gene functions to C level, rather than taxa directly^18,19^, and during phyla-level taxonomic investigations of Bahram et al.^19^ observed stronger correlations between bacteria than fungi for C, our findings are in line with this. A limitation of our metagenome taxonomic profiling approach is the reliance on reference databases, where fungal sequences have historically been lacking, which can make analysis susceptible to false positives. In fact, our observation here is the possible under-representation or diminished predictivity of fungi (potential false negatives) in ML models. It is possible that the use of more comprehensive databases as they become available would allow the inclusion of more of the fungal community, refine fungal species assignment, and improve ML model performance.

### Moving beyond the most predictive features

**Fig. 3:**
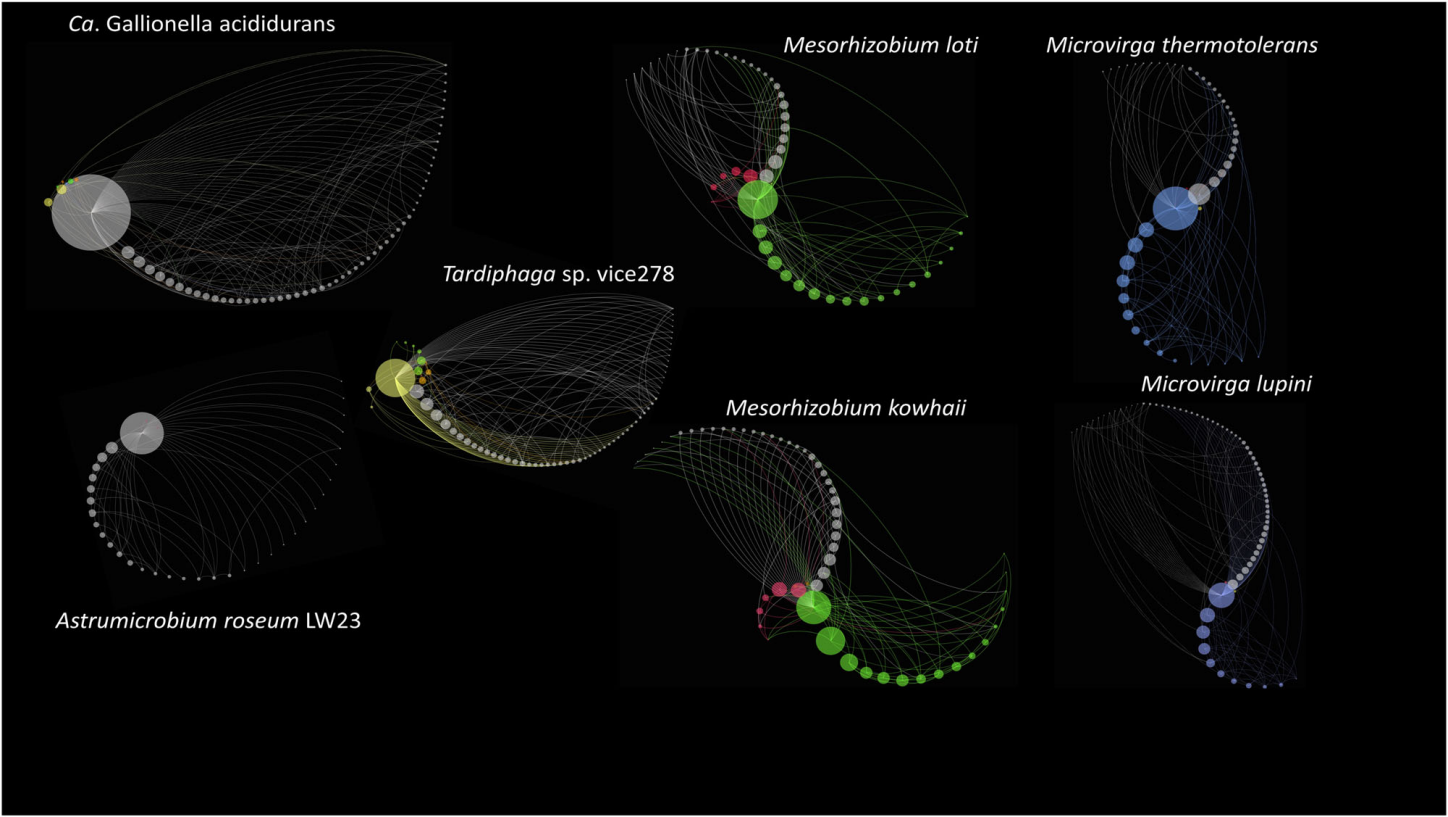
Directed co-abundance networks for individual species identified by SVM as important in predicting SOC content. Sub-networks were extracted from a database-wide, 91,838-taxon co-abundance network generated using SpiecEasi (see “Methods” for details). Sub-networks are shown for all neighbours of *Ca*. Gallionella acididurans, *Astrumicrobium roseum* LW23, the Nitrobacteraceae (yellow nodes) *Tardiphaga* sp. vice278, the Phyllobacteriaceae (green nodes) *Mesorhizobium kowhaii* and *Mesorhizobium loti—*all identified as positive predictive features for SOC*—*and the Methylobacteriaceae (blue nodes) *Microvirga lupini* and *Microvirga thermotolerans*, both identified as negative predictive features for SOC. *Bradyrhizobium* (Nitrobacteraceae) are represented by brown nodes, all other Hyphomicrobiales are represented by red nodes. For each predictive taxon, a radial spiral axis layout was employed to represent the sub-networks with members of the order Hyphomicrobiales listed above represented on separate axes. All other taxa (grey nodes) were allocated to a common axis. Nodes, representative of taxa, were scaled to reflect out-degree, the number of edges going out form that taxon to another taxon. Edge colour reflects the taxonomic association of the target node.

We noted that predictive bacteria may serve as proxies for other species, even though they may not contribute to sequestration directly. To test this and to expand our focus from only the twenty predictive taxa, we derived a database-wide taxon association network using SpiecEasi and extracted association sub-networks of the most predictive bacterial species *Ca*. Gallionella acididurans, *Astrumicrobium roseum* LW23 as a representative of the predictive Verrucomicrobiaceae, and several Hyphomicrobiales chosen for their contrasting SHAP values (Fig. 3a): the Phyllobacteriaceae *Mesorhizobium loti* and *Mesorhizobium kowahii* and the Nitrobacteraceae *Tardiphaga* sp. vice278, all associated with high SOC; and the Methylobacteriaceae *Microvirga lupini* and *Microvirga thermotolerans*, associated with low SOC. The extracted association sub-networks are shown in Fig. 3.

As might be expected for the most predictive taxon, the co-abundance network of *Ca*. Gallionella acididurans was the most extensive and connected (average weighted degree, 1.1; graph density, 0.36). The association network was dominated by *Ca*. Gallionella acididurans and associations between other taxa within the network were limited. The equivalent network for *Astrumicrobium roseum* LW23, although less extensive (average weighted degree, 0.78) was more dominated by *Astrumicrobium roseum* LW23, (graph density 0.05) suggesting that the Verrucomicrobiaceae may form more stochastic associations with other taxa in global soils than *Ca*. Gallionella. Other predictive Verrucomicrobiaceae, *Verrucomicrobium* sp. GAS474 and *Ca*. Verrucococcus diazotrophicus Tous-C9LFEB, were present in this network, as was the ericoid mycorrhizal fungus *Hyaloscypha hepaticola*.

Our analysis (Supplementary Note 5) indicated that co-abundance between Hyphomicrobiales is consistent between soils from widely differing geographic locations and that the taxa are part of closely associating clusters. In contrast to *Ca*. Gallionella and *Astrumicrobium roseum* LW23, *Tardiphaga* sp. vice274, the Phyllobacteriaceae *Mesorhizobium loti* and *Mesorhizobium kowahii*, and the Methylobacteriaceae *Microvirga lupini* and *Microvirga thermotolerans*, are all found in consistent clusters in global soils, predominantly forming congeneric associations. A noteworthy aspect of the *Mesorhizobium* (predictive of high SOC) and *Microvirga* (predictive of low SOC) co-abundance networks is the strikingly similar network topologies between congeners. This is remarkable, given that of the eighty-three taxa in the *Mesorhizobium* networks, only two taxa (*Mesorhizobium metallidurans* and *Mesorhizobium* sp. STM4661) were common to both. In the *Microvirga* networks, only four (*Microvirga calopogonii*, *Microvirga flocculans*, *Microvirga* sp. BT688 and *Tardiphaga* sp. vice154) of eighty-eight taxa were common to both. In both cases, the topology of the co-abundance networks is more conserved than the taxa participating in them.

Given that *Mesorhizobium* and *Microvirga* form associations with plant roots^34–36^ it is possible that these predictive taxa reflect the distribution of plant species across different habitats rather than associations with SOC stocks directly. The abundance variations of *Mesorhizobium* species were most indicative of Boreal and temperate deciduous biomes; while *Microvirga* species were more indicative of Mediterranean, savannah and dry tropical forest biomes (Supplementary Fig. 12). However, we observed that the three *Microvirga* genomes (predictive of low SOC) available on the KEGG GENOME database (at: https://www.genome.jp/kegg/, including *Microvirga thermotolerans*) lack a complete oxidative phase of the pentose phosphate pathway. This pathway is complete in the *Mesorhizobium* genomes available (predictive of high SOC). This suggests that the metabolism of pentoses may be challenging for *Microvirga* species. The pentoses xylose and arabinose are primary monomer subunits of the heteropolysaccharide xylan, which represents the majority of hemicelluloses on Earth, and 20–40% of total plant biomass^37^. Moreover, the pentose phosphate pathway is an autotrophic pathway thought to be responsible for carbon fixation in a range of bacterial species^38^. Therefore, it may be hypothesised that the metabolic capabilities of the species determine their distribution within global soils. To address this hypothesis, we developed an approach to combine species and function information to determine whether this improves predictions of SOC and the insight gained from the modelling.

### DGCNNs improve insight into SOC by combining taxonomic and functional evidence

The graph-based logic of ML can be exploited to associate microbial taxa with functional predictions derived from their genomes. This novel approach was adopted to visualise taxon-function linkages using unique per-sample graphs. Nodes represented microbial species–function combinations, while edge weights represented the number of sequencing reads corresponding to both species and function (or gene) per sample. Additionally, nodes representing sample habitat were included, linked to environmental features (soil water content and air and soil temperatures). In these cases, edge weights represented the environmental feature value. These heterogeneous graphs were used to train a DGCNN (see “Methods”) to predict sample SOC as a graph regression task. As previously, while training our model, the dataset was split into training and held-out test data, and a fivefold CV was performed.

The relatively small number of samples in this study may impede a complex approach. Therefore, we compared the performance of a range of progressively smaller feature sets to derive input graphs. Initially, we trained a DGCNN using the full feature set of microbial species, functions, and environmental data (denoted “Large-graph” with up to 26,138 nodes and 367,428 edges per sample). We next reduced the microbial species to only those within genera represented by the features in Table 1 (thus reducing microbial functions to only those linked to these species) (denoted “Mid-graph” with up to 19,215 nodes and 130,816 edges per sample). Finally, we compared both previous approaches to reducing the microbial species to only the features in Table 1 directly to derive input graphs. In this instance, the nodes encompassed the environmental features plus species and any associated functions that reads aligning to these taxa also mapped to (denoted “Small-graph” with up to 282 nodes and 544 edges per sample).

**Fig. 4:**
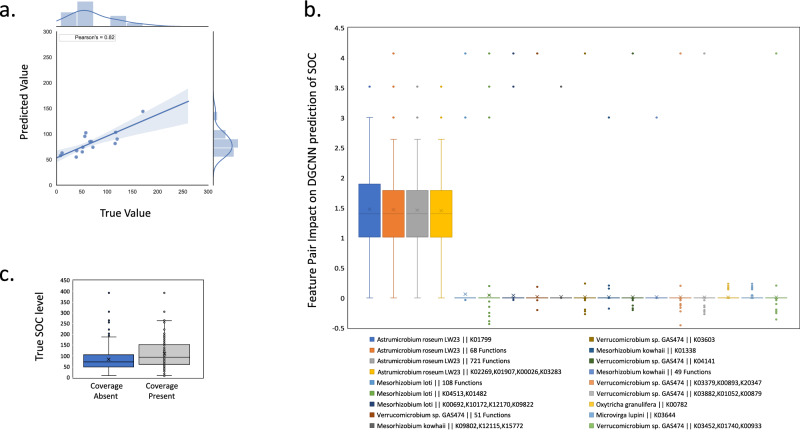
DGCNN models for prediction of SOC. **a** Plot shows the measured (True) versus predicted values for SOC levels (g kg^−1^) for each of the soil metagenomic samples from the held-out test dataset for the “best” DGCNN, i.e., the one trained on the “Small-graphs”. The Pearson’s correlation coefficient (*r*) is also shown (0.82). **b** Box plot to show our calculated species–function node pair “impact values” on the DCGNN model SOC predictions across each of the soil samples. Species–function node pairs are detailed in the legend in the format: species || function (or multiple functions if they share the same mean impact on the sample set). These impact values are used here as a representation of species–function node pair importance (See “Methods” for details of the calculations). Here only the impacts are shown for those species–function node pairs where the mean impact across the samples deviated from zero. **c** For the 69 functions associated with species *Astrumicrobium roseum* LW23 (making up the leftmost boxes from (**b**), i.e., the top two most impactful species–function node pair groups), this box plot compares the depth of sequencing coverage (present/absent) for each of the 69 species–function node pairs in comparison to the real SOC levels across the samples.

Figure 4a and Table 2 provide an overview of the performance of each DGCNN. The “Small-graph” approach demonstrated the most promising results, with a MAE of 34.1 on CV, an MAE of 33.9 on the training data and 27.2 on the test data (RMSE of 30.6). The performance of the DGCNN during CV was comparable to that of the SVM model developed previously (MAE of 32.1). The DGCNN’s CV error amounted to 8.9%, while the SVM error was 8.4%. However, importantly, none of the DGCNNs exhibited signs of overfitting. Although the performance of the SVM and our DGCNN is comparable, the DGCNN has the potential for a more comprehensive representation of microbial communities, providing more detailed insights into the relationship between microbial communities and SOC, without a loss in model performance. Since the largest graphs performed less effectively, it is possible that we are unable to exploit their high complexity with our small sample number. However, with a larger cohort, these graphs may generalise better, explaining more of the variation between different soils. Nevertheless, the model performance was excellent on the test data indicating that the “Small-graph” was sufficient for learning.

**Table 2:** Comparing DGCNN models for prediction of SOC

Estimator	Large-graph	Mid-graph	Small-graph
**Test MAE**	41.615	41.518	27.194
**Train MAE**	41.589	40.574	33.914
**Mean MAE CV**	41.416	41.841	34.081
**SD MAE CV**	3.638	5.702	3.540

DGCNN model performance comparison between our “Large-graph”, “Mid-graph” and “Small-graph” examples for a range of metrics including Mean Absolute Error (MAE) on the training dataset (Train MAE), MAE on the held-out test set (Test MAE), the mean and standard deviation of the MAE value after five-fold CV (Mean MAE CV, SD MAE CV).

To derive meaningful insights from the DGCNN, we must understand which species–function combinations or environmental features influence the predictions of SOC. We derived a method analogous to permutation importance for node pairs, ranking their relative impact on the prediction of SOC per sample (see “Methods”). To assess the impact of a change in taxon *x* or function *y* on the predicted SOC level, we employed this permutation method to compare the influence of a maximum and minimum node pair edge weight on the predicted SOC level per sample (see “Methods”). In a scenario where identical taxa have similar genome lengths across the soils, then all gene lengths and thus coverages derived from metagenomic data will be contingent upon species abundance across the samples. Consequently, all functions associated with a species would be equally predictive. However, in global soils exposed to a wide range of stresses, we anticipated that significant microbial adaptation would be evident, for example, via genome streamlining and horizontal gene transfer. Consequently, differences in impact between node pairs, e.g., between pairs from the same species in a sample or between samples, could signify changes in the genome sequence of a species. These changes could be identified by comparing the normalised gene coverage differences in our metagenomic data between soils. This approach could provide a powerful tool for identifying microbial adaptation to different environments.

Our permutation importance approach was employed to assess the impact of node pairs in our DGCNN at a global level across the samples. Figure 4b illustrates this global analysis for the 1025 taxon–function node pairs from six taxa where mean impact was non-zero. This suggests that multiple functions from a small set of taxa had high predictive power. The most predictive node pair was the function K01799 (representing maleate isomerase EC:5.2.1.1 genes *nicE* and *maiA*) from the plant root-associating methylotroph^39^ *Astrumicrobium roseum* LW23. This taxon-gene combination is predicted to be rare: Using AnnoTree version 95^40^ to observe the taxonomic distribution of K01799 indicates that the gene is present on 4314 (2.3%) of 191,527 bacterial genomes represented in the Genome Taxonomy Database but that it is present on only three Verrucomicrobiota genomes. The enzyme catalyses the interconversion of maleate and fumarate during nicotinate and nicotinamide metabolism, which itself forms part of the metabolism of cofactors and vitamins. It also participates in butanoate metabolism, part of wider carbohydrate metabolism (Supplementary Fig. 13). Verrucomicrobiaceae ecology is poorly understood, but the group appears to be numerous in soil bacterial communities across the globe^41^ where they are thought to play important roles in carbohydrate turnover, (poly)saccharide degradation^39^, and methane oxidation. The group shows a preference for carbohydrates and is capable of xylan, chitin, or cellulose degradation. It is thought that only a limited range of substrates are utilised as sole carbon and energy sources, mainly hexoses, di- or trisaccharides or glucose derivatives^39,42,43^(Table 1). The closely related *Verrucomicrobium* sp. GAS474 is also identified as an important feature in Fig. 4b, consistent with our previous observation of three predictive Verrucomicrobiaceae from the classic ML analysis.

Figure 4b groups taxon-function node pairs according to a conserved taxon node and a conserved mean impact across the sample set, resulting in four groups of most predictive node pairs. All of these node pairs were associated with *Astrumicrobium roseum* LW23 (average impact across the samples ranging from 1.48 to 1.45). The next fourteen node pairs were derived from Hyphomicrobiales, namely *Mesorhizobium loti, Mesorhizobium kowhaii* and *Microvirga lupini*, all commonly isolated from root nodules of leguminous plants^30,44,45^; from *Verrucomicrobium* sp. GAS474, originally isolated from forest soil of a long-term soil warming experiment^46^ and closely related to *Astrumicrobium roseum* LW23^39^; and from the hypotrich ciliate *Oxytricha granulifera*, which is broadly distributed in global soils^47^. All these taxon-function features showed a consistent but significantly reduced average impact across the samples, ranging from 0.06 to 0.01, respectively.

In Fig. 4b, multiple functions from each taxon were equally predictive, as anticipated. Notably, not *all* functions from every taxon were equally predictive, indicative of biogeographic differences in genomes between samples. With the depth of sequencing coverage available, taxon-level per-sample genome assemblies were not possible. Instead, in Fig. 4c, to account for differences in sequencing depth, we compared the normalised sequencing coverage with SOC levels across the samples for the most discriminatory sixty-nine taxon-function node pairs that positively impact SOC prediction. Samples with coverage of these functions had a significantly higher SOC level (mean 110 g kg^−1^) compared to samples with no coverage of these functions (mean 84 g kg^−1^) (two-tailed *t*-test, df = 1311, *t* = 1.96, *p* < 0.001). Piton et al.^18^ proposed that genome streamlining by gene loss is associated with low soil C:N ratios. Our observations linking coverage loss to lower SOC levels for highly predictive species-level functions are consistent with this hypothesis.

We explored this further using sample-specific local investigations of the two samples compared previously (sample S234 with high SOC and S72 with low SOC, Fig. 2b, c). For these two samples, we used our permutation importance approach to investigate the impact of node pairs for SOC predictions (Supplementary Note 6; Supplementary Fig. 14a, b). This DGCNN analysis revealed that predicted SOC levels for the two samples were more closely aligned with measured levels than those based upon the previous classic ML analysis with SVM. Prediction of SOC for sample S234 (high carbon) was sensitive to both positive and negative impact node pairs, the majority of them associated with Verrucomicrobiaceae (Supplementary Fig. 14a). This analysis provides examples of when different functions from the same species could be unequally predictive for a given sample. By comparison, S72, a lower carbon sample (Supplementary Fig. 14b), had only positive impact node pairs affecting its prediction.

Verrucomicrobiaceae are commonly identified in soil environments. They are recognised for their ability to degrade complex organic compounds^48^. In addition to C cycling, they are thought to be important for soil nutrient cycling, particularly nitrogen cycling. Several increasingly complex models (Random Forest, SVM and DGCNN) identified Verrucomicrobiaceae features as important in the prediction of SOC in globally distributed soil samples. In addition, using a DGCNN, a number of genetically coded functions associated with these Verrucomicrobiaceae are identified as both important positive and negative features for SOC prediction. Verrucomicrobiaceae exhibit a broad range of genome sizes^39,49^ with evidence of horizontal genetic transfer^50^. The identification of several genome-encoded functions of these organisms as being both positive and negative predictors of SOC possibly reflects a degree of this genome flexibility and environmental selection of functions. This inference warrants further investigation. However, the consistent identification of this group of organisms as predictors suggests the global importance of Verrucomicrobiacae for C cycling in soils.

## Discussion

A novel approach to modelling microbial communities was developed by combining microbial taxa and their genetically coded functions in a predictive DGCNN. This approach was used to predict SOC levels using graph-based representations for each soil microbiome. The incorporation of these representations facilitated a more precise depiction of microbial characteristics in the model, thereby enhancing the predictions of SOC. The DGCNN distinguished between the functions of individual taxa. We have interpreted this differentiation as reflecting differences in species’ genome sequences between samples. One potential explanation for this phenomenon is the loss or acquisition of environmental genes. However, to confirm this hypothesis, we require a greater depth of sequencing coverage to allow assembly to generate Metagenome-Assembled Genomes (MAGs) and examination of sequence differences in the most predictive features of the model. The generation of MAGs (and their subsequent functional annotation) could also be used as an alternative approach to generate input information for our DGCNNs to potentially overcome limitations that our approach may encounter from incomplete taxonomic classification databases. In addition, we devised a method for DGCNN interpretation, generating biological perspectives into the most informative taxon-function pairs for SOC prediction. Multiple predictive taxon-function pairs were associated with Verrucomicrobiaceae and carbohydrate metabolism, implying that this relatively understudied but widespread family plays an important role in SOC dynamics globally. The identification of biologically significant taxa and their associated functions can help generate hypotheses for future experiments regarding microbes that have an impact on the global C cycles.

## Methods

### High-level summary of the analytic process detailed in subsequent methodology subsections

A summary of the methodology/workflow presented in this study is provided in Supplementary Fig. 15. Throughout the manuscript, we follow a process that includes

1. Sample processing (e.g., via a multi-software bioinformatics workflow)
2. Comparison to derive the most effective normalisation approach (e.g., for microbial species and functional abundance tables)
3. Feature integration (e.g., via tabular concatenation or developed graph structures)
4. Classic ML (e.g., involving training and testing a series of ML regressors, optimising each regressor model over a range of hyperparameters, followed by selection of the best-performing model per feature set as proposed in ref. ^51^)
5. Feature selection (e.g., freezing the best-performing classic ML model and sequentially reducing input features for training)
6. DL via a DGCNN (e.g., where each sample forms an input graph connecting species to functional abundances, this DGCNN refers to a single model architecture)
7. Model explainability/interpretability (e.g., using SHAP for classic ML and a bespoke method for the DGCNN to identify top predictors)
8. Generating co-abundance networks for predictive features to explore hidden links

All ML/DL tasks refer to the prediction of SOC level. Importantly, there are commonalities for both the classic and graph ML workflows where our final best models require normalised, pre-processed input data and conserved partitioning of the input dataset into train and test sets across all analyses. Conserved partitioning of the data into training and test was achieved by using the “random_state” function in scikit-learn to determine the splitting of data into train and test indices; this function was also used during 5-fold cross-validation to ensure reproducibility of partitioning for cross-validation.

Eighty-five percent of the data was used for training, the remaining 15% was held out for testing, and five-fold cross-validation was performed on the training data using *K*-folds with a Random state 40.

### Soil samples

We used 189 metagenomic samples, covering all terrestrial regions and biomes of the world, generated, presented and discussed in the study by Bahram et al.^19^. The Bahram et al.^19^ study sampled 58,000 top soils (5-cm diameter soil cores to a depth of 5 cm) from 0.25-ha plots (40 subsamples per site) at 1450 sites, harbouring homogeneous vegetation that was minimally affected by humans. They minimised biases and shortcomings in sampling as well as technical variation, including batch effects, by using highly standardised collection and processing protocols. From the total collection, a subset of 189 representative high-quality DNA samples were chosen for whole shotgun metagenome analysis (of which 184 passed quality filters for inclusion in this study), they spanned different vegetation types (including forest, grassland and tundra biomes) separated by spatial distances that were sufficient to minimise spatial autocorrelation and to cover most areas of the globe (for more details, see original publication^19^ and its Extended Data-Figure 1a for image of sample geographical locations). The sequence datasets were comprised of paired-end reads, each of 250 bp.

Bahram et al.^19^ noted that predictions may be limited by the vast diversity in soil microbiomes, e.g., local variation in environmental conditions such as pH may lead to deviation from general patterns. They proposed that the large spatial range and strong environmental gradients covered in their sampling design, together with the long-term persistence of DNA in soil, would minimise the impact of seasonal variation. In addition, most samples were collected during the vegetation growing season, further reducing seasonal biases. They tested the effect of sampling month and season, finding no significant effect of seasonality on diversity indices. They also compared the effect of seasons and years in a time series study at two sites, which revealed no seasonal effects on richness and composition. We investigated these assertions after analysis of the same sample set with our comprehensive bioinformatics workflow—we assessed temperature seasonality (defined as the standard deviation of monthly temperature averages over 20 years), where we found significant associations between temperature seasonality and the alpha diversity metrics species richness (*r* = −0.215, *p* = 0.004), Chao1 (*r* = −0.216, *p* = 0.004), evenness (*r* = −0.203, *p* = 0.007), Shannon index (*r* = 0.163, *p* = 0.031) and Simpson index (*r* = 0.172, *p* = 0.022) as per previous studies^52^.

### Bioinformatic analysis workflow

We developed a bioinformatic analysis workflow for the taxonomic and functional annotation of paired-end reads (Supplementary Fig. 16). Our workflow was used to process the metagenomic sequence data from the 189 samples to reveal microbiome taxonomic composition. We also assessed functional potential by determining and tracing gene family abundances, which may be involved in various functional pathways, where we assume correspondence between gene functional potential and the resulting ecosystem functioning or enzyme activities. Our workflow is intentionally redundant in order to annotate as many reads as possible, i.e., considering multiple software for both taxonomic and functional annotation. Software was selected based on coverage of different methodologies, level of usage and its likelihood to give us the best performance for a set task. As an example, in previous work, we found Kraken2^53^ typically uniquely “classified” a larger proportion of reads with the highest speed and lowest memory requirements, while DIAMOND ran slower and “aligned” fewer reads but with greater precision^20,54^.

In our workflow, we first performed read quality control. Using Trimmomatic v2.9^55^, reads were trimmed of adaptor and also trimmed using a sliding window approach; if the average quality across a 4 bp window was less than 15, reads below 40 bp were dropped from the analysis. Those read pairs that remained were processed by FastUniq v1.1^56^ to remove duplicates (default settings). As an additional optional step, the quality-filtered read pairs were merged using FLASH v1.2.11^57^, designating an average read length of the sample set of 250 bp, a fragment length of 350 bp and a read length standard deviation of 30 bp.

Following quality control, reads were processed using a taxonomic profiling workflow (Supplementary Fig. 16, purple). The NCBI-nr protein database (downloaded 07/09/2021) was used for all taxonomic analysis, i.e., used to build custom databases for alignment/classification using default settings for DIAMOND/Kraken2 makedb and build commands, respectively. Quality-filtered read pairs were input directly into Kraken2 v2.1.2^53^ for read classification (using default settings) against the NCBI-nr database. For these classified reads, Bracken v2.6.2^58^ was used to calculate species-level abundance estimates, where a minimum number of ten reads was required for classification at a specific rank. In parallel, both the merged and unmerged quality-filtered reads (after processing with FLASH) were used as input into DIAMOND v2.0.11^54^ for read alignment in blastx mode using “-b 25 -k 5 –index-chunks 4 –min-score 50 the –more-sensitive flag and –max-target-seqs 2” options. This resulted in an e-value cutoff of approximately 1e-5. For these DIAMOND aligned reads, the suite of tools from the MEGAN community edition v6.21.12^59^ was used to run a last common ancestor (LCA) analysis to allow binning of reads by taxon, i.e., species-level assignment (here a single assigned “best hit” taxonomy is derived per read pair if there were two unmerged paired query reads, or if there were multiple hits for a merged read pair). By default, MEGAN performs taxonomic binning by assigning reads to nodes in the NCBI taxonomy using the LCA algorithm. Finally, the “daa2info” function of MEGAN was used to also extract functional alignment information for the KEGG (Kyoto Encyclopaedia of Genes and Genomes) functional classification scheme^60^. MEGAN outputs were then processed using custom Perl scripts to extract and compute species-level abundance (read counts) and KEGG orthologue abundances. Supplementary Table 7 details the output files/tables from these analyses.

In parallel to the taxonomic profiling workflow, the reads that passed quality control were input into a third functional profiling workflow (Supplementary Fig. 16, pink), with the aim to describe the metabolic potential of each microbial community. This workflow acts as a supplement to our previous DIAMOND-MEGAN functional annotation and uses nucleotide mapping and translated search to provide organism-specific gene and metabolic pathway abundance profiles for each analysed metagenome. For this functional profiling workflow, the reference database used for comparison was the NCBI-Uniref50 protein database (downloaded on 08/09/2021), which is recommended for analysis of diverse microbiome, such as those originating from the soil, alongside the requirement for reads to map at 50% identity. Both merged and unmerged quality-filtered reads (after processing with FLASH) were used as input into HUMAnN 3 v3.0.13^61^. The HUMAnN 3 standard workflow was run encompassing; taxonomic profiling (MetaPhlAn3), read alignment (Bowtie2/DIAMOND) and pathway/gene family abundance and coverage calculations for the KEGG functional classification scheme^60^. We then used custom Perl scripts where necessary to extract and compute KEGG orthologue abundances.

### Bioinformatic workflow outputs

The output files from the developed bioinformatic workflow are summarised in Supplementary Table 7. From these outputs, we created a series of read count matrices (csv files) where the 189 samples were added as rows, and the columns detailed the number of read pairs per sample aligned to each individual species (taxonomic profile) or KEGG number (functional profiling). For comparison, read count matrices were generated separately for species using; Kraken2 (189 samples × 83,333 species), Kraken2 after Bracken correction (189 samples × 27,399 species) and Diamond (189 samples × 48,952 species). Additionally, read count matrices were generated separately for KEGG functions using; Diamond (189 samples × 100,147 functions) and HUMAnN 3 (189 samples × 17,438 functions). Supplementary Table 8 summarises the overlap and main sources of information that were generated from each section of the workflow.

We next combined the multiple taxonomic annotations to create a single taxonomy matrix, we also did this for the multiple functional annotations to create a single matrix associated with function. To avoid redundancy, e.g., counting the same read mapped by Kraken2 and Diamond twice, we defined the most confident or best mapping for each read. All tools provide (or give the means to calculate) the length of the read that mapped to each species or function it has been assigned to, this gave us a metric to compare between different software. The longest taxonomic and/or functional mapping was selected for each read pair to ensure that each read pair was only counted once during the generation of our taxonomic and functional read count matrices (where mapped lengths were equal between multiple tools, we defaulted to Diamond). Using this method, we were able to use the output of one mapping approach to fill in blanks from another, to annotate as many reads as possible. Our final “combined” matrix for taxonomy encompassed mapping by Kraken2 and Diamond, resulting in 189 samples with read counts across a total of 91,838 species. Our combined matrix for function encompassed mapping by Diamond and HUMAnN3 resulted in 189 samples with read counts across a total of 100,147 KEGG numbers representative of functions.

### Data normalisation and diversity estimates

To allow comparison between samples, with our primary aim being analysis using machine learning (ML), all abundance matrices were normalised to the total number of reads used for mapping unless otherwise specified below. This accounts for different sequencing coverages (library size) and includes the fraction of unmapped reads.

All statistical analyses relating to taxonomic diversity were performed using R packages (v4.0.2)^62^ unless otherwise stated. We removed 5 samples (G2754, G2839, G2645, G2649, S36) that had counts of species richness that fell significantly below the other samples and would be detrimental for rarefication (Supplementary Fig. 17a), we also removed these samples from all subsequent analyses including ML leaving 184 samples for analysis. For the taxonomic abundance matrices, we first generated a species accumulation curve using the function *specaccum*, from the package *vegan* (v.2.5.7; https://cran.r-project.org/web/packages/vegan/index.html) (Supplementary Fig. 17b). We next generated a rarefaction curve using the *rarecurve* function from *vegan*, which shows the expected number of species represented in *n* individuals drawn at random from the large pool of *N* individuals (Supplementary Fig. 17c, d). For the taxonomic read count matrices, species abundances were rarefied to an equal number per sample to reduce the effect of variation in sequencing depth using the function *rrarefy* from *vegan*.

We next calculated a range of alpha diversity metrics to summarise the structure of our microbial communities and, where appropriate, compared raw taxonomic species abundances to rarefied counts, that correct for the lack of sampling standardisation. We calculated the following measures (using functions from *vegan*): observed species **richness** per sample^63^ (function *specnumber*); **Chao1**^64^ nonparametric lower bound estimate of species richness (function *estimateR*); Pielou’s **evenness**^65^ (function *diversity*); **Shannon index**^66^ (function *diversity*); **Simpson index**^67^ (function *diversity*). We used analysis of variance (ANOVA) to assess if the diversity means were different across habitat groups, and we used general linear models to assess mixed or continuous variables including geographical locations.

For our calculated alpha diversity metrics including evenness, Shannon and Simpson diversity indexes, we observed minimal effect when comparing the species abundance counts that were rarefied or raw (Supplementary Fig. 18; Supplementary Table 9). This was evidenced by highly correlated (*r* ≥ 0.982, *p* < 0.00001) values. Based on these results, we used the metrics evenness, Shannon and Simpson diversity indexes as calculated from raw counts for inclusion in downstream ML analyses. Furthermore, the original study that developed the metagenomic samples that we are using^19^ noted a high correlation between taxonomic diversities calculated using Simpson and Shannon diversity (*r* = 0.888), which we also observed here with (*r* = 0.889, *p* < 0.00001) and without (*r* = 0.888, *p* < 0.00001) rarefying the species abundance counts. In contrast, observed and estimated species richness values were more affected by rarefication (*r* ≤ 0.261) (Supplementary Fig. 18; Supplementary Table 9). For the raw counts, sample richness for most samples fell between 20,000–40,000 species, while the Chao1 estimated an overlapping but slightly increased richness for the majority of samples between 25,000 and 50,000 species suggesting, that sequencing depth was suitable for many samples but not quite enough to catch all the diversity for all samples. The correlation between richness and Chao1 was also strong with (*r* = 0.876, *p* < 0.00001) or without (*r* = 0.994, *p* < 0.00001) rarefying the species abundances.

For comparison, we used the R package *iNEXT* to calculate Hill numbers of order *q*: species richness (*q* = 0), Shannon diversity (*q* = 1, the exponential of Shannon entropy) and Simpson diversity (*q* = 2, the inverse of Simpson concentration). For each diversity measure, we used *iNEXT* to compute diversity estimates and associated 95% confidence intervals using 399 bootstraps. We used *iNEXT* to plot first, sample-size-based R/E sampling curves, i.e., of diversity estimates with respect to sample size, where diversity estimates are computed for rarefied and extrapolated samples up to double the reference sample size. Second, we generated coverage-based R/E sampling curves of diversity estimates for rarefied and extrapolated samples with sample completeness up to the coverage value of 25 million reads (our largest sample size). We also generated sample completeness curves to show how the sample coverage estimate varies as a function of sample size.

We used the rarefied species abundance counts to investigate beta diversity, or the microbial taxon composition difference between samples using *Bray-Curtis* dissimilarity that considers species occurrence data directly (abundance), and also the *Jaccard* distance that is based on presence/absence species information data. In both cases, 0 infers samples sharing the same species, and 1 means that samples have no species in common. We plotted beta diversity using ordination to capture the many dimensions (species) in fewer “artificial” dimensions in which similar species and samples are plotted close together, and dissimilar species and samples are placed far apart. First, we used Principal coordinates analysis (PCoA) to represent the distances between samples in a low-dimensional, Euclidean space. PCoA maximises the linear correlation between the values in the dissimilarity matrix (we used *Bray-Curtis*). Non-metric Multidimensional Scaling (NMDS) rectifies the linear assumption of PCoA by maximising the rank order correlation. As such, next, we used NMDS to represent the pairwise dissimilarity between objects in a low-dimensional space (also based on *Bray-Curtis* dissimilarity). We observed similar trends for *Jaccard* distance and *Bray-Curtis* dissimilarity (Supplementary Fig. 19). As such, for downstream investigation, we focused on *Bray-Curtis* dissimilarity since it considers species abundance. Finally, we performed a permutational multivariate analysis of variance (*PERMANOVA)* statistical test using the *adonis* function in vegan (using 9999 permutations for the pseudo-*F* test statistic and its statistical significance) this compared distances of samples within the same group to distances of samples from different groups across habitats and geographical locations. This was applied with the Bray-Curtis dissimilarity measure.

We compared *Bray-Curtis* dissimilarity and *Jaccard* distance to Unifrac distances (weighted and unweighted) that incorporate phylogenetic distances. To calculate Unifrac distances, we used the *classification* function from the R package *taxize* to retrieve the taxonomic hierarchy from NCBI for each species in our abundance matrix (12,144 species with a read count of ≥10 in ≥10% of samples). We then used the function *class2tree* to convert these NCBI classifications to a tree with weighted branches (*phyloseq* phylogenetic tree object) that we use for UniFrac calculations. We compared weighted unifrac (wunifrac) and unweighted (unifrac) with/without rarefying the taxa.

### Environmental data analytics

Environmental data matching the geo-locations of each of the metagenomic soil samples was sourced from three different input datasets covering soil properties and weather data (Supplementary Table 5). All environmental data was pre-processed and imported into the IBM Environmental Intelligence Suite^26,27^, a scalable geospatial data storage system enabling large-scale data exploration and gathering for AI workflows. The use of this platform allowed easy and rapid data querying for all field soil sample locations. Soil data was gathered from the global 250 m resolution SoilGrids dataset^68^. The dataset provides details on ^69^; volumetric fraction of coarse fragments (we denote as “course_depth”), soil organic carbon (“orgcarbon_depth”), sand (“sand_depth”), clay (“clay_depth”), silt (“silt_depth”), nitrogen (“nitro_depth”), pH (“pH_depth”), organic carbon density (“carbondens_depth”), bulk density of fine earth fraction (“bulk_depth”), cation exchange capacity (“cation_depth”) and soil class (“soilclass”). For some variables, there are six soil depth intervals available: 0–5 cm, 5–15 cm, 15–30 cm, 30–60 cm, 60–100 cm, and 100–200 cm, we focused on 0–5 cm to be consistent with the sampling depth for the metagenomic samples. Quantile refers to the output of the quantile regression forests that SoilGrids uses as its predictive model. We retrieved the 5%, 50% and 95% quantiles as well as the mean and uncertainty under the quantile dimension. The mean was used for predictions of the soil property. The mean represents the ‘expected value’ and provides an unbiased prediction of each soil property. The 5% and 95% quantiles represent the lower and upper bounds of a 90% prediction interval and we have used these as a measure of prediction uncertainty. The SoilGrids dataset is the output of a ML model, hence we have some measurements as a mean value but also the quantiles as well.

Weather data was derived from Global weather (ERA5) Layers from 1980-01-01 to 2021-10-19, these include^70^; soil water at 0 to 7 cm (“soilwater0to7cm”), soil temperature at 0 to 7 cm (“soiltemp0to7cm”), total precipitation on the day of sampling for metagenomics (“totalrain”), temperature on day of sampling for metagenomics (“temp”), year and month of sampling for metagenomics, type of low vegetation and type of high vegetation. The ECMWF model considers the following ten types of vegetation as low^1^: 1 = Crops, mixed farming^1^ 2 = grass, 7 = tall grass, 9 = tundra, 10 = irrigated crops, 11 = semidesert, 13 = bogs and marshes, 16 = evergreen shrubs, 17 = deciduous shrubs, 20 = water and land mixtures. The ECMWF model considers the following ten types of vegetation as high: 3 = Evergreen needleleaf trees, 4 = deciduous needleleaf trees, 5 = deciduous broadleaf trees, 6 = evergreen broadleaf trees, 18 = mixed forest/woodland, 19 = interrupted forest. Others: 8 = desert, 12 = ice caps, 14 = glaciers, 15 = inland water and ocean. Weather data is available hourly but then resampled to give the monthly average for the last 20 years (for precipitation, temperature, soil temperature and soil water these were denoted as “totalrain_long”, “temp_long”, “soiltemp_0_7_long” and “soilwater_0_7_long” respectively). This provides a good overview of the general climate for each specific location. Further, the climate variable was taken for a specific location on the day the sample was taken to get an understanding of the conditions on a specific day. Since the metagenomic soil samples were taken at the topsoil level (top 5 cm), here we use the soil information from the 0–7 cm depth initially. For the weather, we focused on the weather conditions on the actual day and then the difference from a 20-year average (for precipitation, temperature, soil temperature and soil water these were denoted as “totalrain_diff”, “temp_diff”, “soiltemp_0_7_diff” and “soilwater_0_7_diff” respectively) to give an indication if the day the sample was taken was in a normal range for that particular area.

As a preliminary approach to investigating general relationships between climate and edaphic variables and the phylogenetic distinctness of soil microbial communities, we used canonical correlation analysis (CCorA) to identify linear combinations of environmental variables and linear combinations of phylogenetic distance between soil microbial communities that were maximally correlated with one another, using weighted UniFrac as the measure of phylogenetic distance. To guard against over-parameterisation of the model, we minimised the appropriate subset of axes (*m*) determined from principal coordinates analysis using a leave-one-out residual sum of squares, choosing *m* associated with the smallest sum of squares. We used the sum of canonical eigenvalues as a test statistic, inferring probability by performing 99,999 permutations and assuming the exchangeability of the samples under a null hypothesis of no differences in the positions of biome centroids in multivariate space. Since the original analysis of taxonomic distinctness between biomes^19^ indicated that bacterial and fungal distributions were sensitive to different environmental variables, we studied the influence of environmental variables upon the phylogenetic distinctiveness of global soil bacterial, archaeal and fungal assemblages separately.

### Preliminary classic ML analytics

In our first ML analysis, we developed trained and tested ML models to compare the effect of different data transformation techniques on predictive capability separately for the taxonomic and functional abundance matrices. These data transformations included: raw abundance counts, rarefied counts (taxonomy only), normalised counts including unmapped reads ([counts per species or function/total number of reads used for mapping] * 1 × 10^6^) and normalised counts using only those reads which were mapped ([counts per species or function/total number of reads mapped] * 1 × 10^6^). The predictivity of the alpha diversity metrics (including Hill numbers) was also tested. As our target variable, we predicted the average soil organic carbon (SOC) content (g kg^−1^) for each location that the soil samples were taken from (depth 0–5 cm), as a regression task. These measurements were derived from SoilGrids, and the SOC levels across the analysed sites are shown in Supplementary Fig. 20.

We used scikit-learn (v3.7)^71^ to build and tune ML models, adopting the following approach: *MinMaxScaler* was used to scale the features from 0 to 1, 85% of the data was used for training, the remaining 15% was held out for testing, and five-fold cross-validation was performed on the training data using *K*-folds. Method hyperparameters were optimised using a grid search, testing a range of parameters (Supplementary Table 10) for the following regressors: Logistic Regression, Random Forest, XGBoost, LightGBM, Support Vector Machine (SVM), Gaussian process, Gradient Boosting and *K* nearest neighbours (KNN). We selected the “best” ML model (using best parameters after fine-tuning), according to the lowest mean absolute error (MAE) on the test data and after cross-validation, moderated by the least overfitting between training and test data (Supplementary Table 11).

Having identified the best transformation technique approach for each of the three feature sets (taxonomic abundance, functional abundance, alpha diversity metrics/Hill numbers), we combined all three feature sets (191,993 features in total across 184 samples) with their determined normalisation and built and tuned the eight regressors using these 191,993 features to predict soil organic carbon content. We identified Random Forest as the “best” ML model (using best parameters after fine-tuning), based upon it having the lowest MAE on the test data and after cross-validation, and the least overfitting between training and test data. We then used *f_regression* univariate linear regression tests (implemented via the scikit-learn’s *SelectKBest* function) to reduce the number of features sequentially, choosing the most positively correlated features with the target for each subset size. We started reducing feature numbers using large increments >10,000, reducing this to increments of 1000, 10 and 1 as we narrowed the window for the most appropriate number of features. Following each reduction in feature number we re-trained and tested our “best” ML model again with cross-validation. This allowed us to identify a set of highly predictive features. Finally, we compared usage of Principal Component Analysis (PCA) for feature selection with *f_regression* univariate linear regression tests; we tested a range of component numbers according to those explaining 0.75 (3 components), 0.80 (4 components), 0.90 (6 components), 0.95 (10 components), 0.99 (25 components), 0.995 (38 components), 0.999 (83 components), 0.9999 (152 components) of the feature set variance. PCA comparisons consistently yielded greater error rates than feature selection with *f_regression*.

### Sparse InversE Covariance estimation for Ecological Association and Statistical Inference

Since the microbiome is an interactive community, our predictive microbial abundance features from the classic ML could be representing larger interactomes or highly correlated/co-abundant species. As such, we used Sparse InversE Covariance estimation for Ecological Association and Statistical Inference (R package SpiecEasi^72^) to generate an overall network for all of our species, then we inferred sub-networks of the closest neighbours for a selection of predictive features of interest. SpeicEasi is designed to accommodate compositional and sparse data typical of metagenomic data. Our method was as follows; we filtered species with abundances of 5 or more in 50 or more samples (leaving 12,902), we then used this filtered abundance table to run Spiec-Easi’s neighbourhood selection method (mb) with a *lambda.min.ratio* of 0.01, *nlambda* of 5, *sel.criterion* ‘bstars’ and *pulsar.params* consisting of 10 replicates and a seed of 10010. Next, we created an iGraph (https://igraph.org) object from the SpiecEasi output, used the *neighbors* function for each of our microbial taxa of interest with mode ‘all’ to extract all connecting taxa to it, used the *induced.subgraph* function to create a subgraph for each taxon of interest. These sub-graphs were visualised in Gephi 0.9.2^73^ using a Radial Axis layout. Genera were assigned to different axes and nodes (representing individual taxa) were scaled according to out-degree.

### Graph-based ML analysis

Given that there is an inherent relation between microbial taxonomy and functional annotation within the context of bioinformatics, we developed a fresh data representation technique that could capture this relationship for each sample in the form of graphs. We then used these graphs to perform a graph regression task to predict the average SOC content for each location that the soil samples were taken from (depth 0–5 cm) (Supplementary Fig. 21a–c). The nodes in a graph consisted of taxonomy and function identifiers, as shown in Supplementary Fig. 21a. The links between the related taxonomy and function ids made the edges in that graph. We first generated “Edge lists” for each of the samples (Supplementary Fig. 21a, b). Each record in these “Edge lists” was in the format “source,target,weight” where the “source” denoted the microbial species, the “target” denoted the KEGG orthologue number, and the “weight” related to the number of pairs of sequencing reads that aligned to both protein sequences representing the “source” and sequences representing the “target” after the DIAMOND alignment. For each sample, there was also a small group of edges representing a subset of our environmental data. Here, the “source” was denoted as the habitat that the sample was derived from, and the “target” was the “temp_long” or “soilwater_0_7_long” (see Environmental data analytics section for details), with the weight being the respective value of the target, i.e., the temperature or soil water level.

Next, we used the Networkx function^74^ to load these edge lists as network undirected graphs (function *read_weighted_edgelist*). We generated 1 graph per sample. Thus, the nodes of the graph became mainly microbial species and KEGG numbers (functions). Example sample graphs for two samples are shown in Supplementary Fig. 21c. Graph node features were added that encompassed labelling of whether a node was a species (denoted 0) or a function (denoted 1) and labelling whether a function was part of the KEGG pathway for central carbon metabolism (denoted 1) or not (denoted 0). Labels for each of the samples were associated with the graphs according to our prediction target, i.e., the average SOC content for the sample collection location. We then used the *from_networkx* function to load the graphs into a list where each graph was formatted for use with *Pytorch Geometric*. Approximately 15% of the sample graphs were selected from the dataset (as per the approach used for the classic ML) and held out for testing. The remaining samples were loaded using the *Pytorch Geometric DataLoader* (shuffle = true) and used for training and testing of the DGCNN.

Our graph-based ML analysis was performed via a Deep Graph Convolutional Neural Network (DCGNN) using *PyTorch Geometric* v2.1.0^75^. This DGCNN uses the graph attentional^76^ and the graph convolutional operator^77^. The architecture for our DGCNN was as follows (Supplementary Fig. 21d);

Layer 1: GATConv(2, 32, heads = 1)

Layer 2: GATConv(32, 32, heads = 1)

Layer3: GATConv(32, 32, heads = 1)

Layer 4: GATConv(32, 128, heads = 1)

Layer 5: GCNConv(128, 128)

Layer 6: AdaptiveMaxPool1d(output_size = 128)

Layer 7: GCNConv(128, 128)

Layer 8: Flatten(start_dim = 1, end_dim = −1)

Layer 9: Linear(in_features = 128, out_features = 32, bias = True)

Layer 10: Linear(in_features = 32, out_features = 1, bias = True)

We optimised the DGCNN architecture, testing a range of different convolutional layer types and combinations. We also tuned parameters including the learning rate, number of epochs and weight decay. After hypertuning, the training and testing of the final DGCNN were performed using 100 epochs, a learning rate of 0.001, weight decay of 0.1 and performing 5-fold cross-validation.

To interpret the results of the DGCNN model, with respect to the most important “source”-“target” edge list pairs for prediction of SOC level we calculated an “impact score” for each “source”-“target” pair (individually). Our method was as follows per “source”-“target” pair; for each sample, we set the pair weight to the lowest observed value across the samples, and we generated a graph from the amended edge list and then predicted the SOC level for it from our trained model. We repeated this exercise for the same sample using instead the highest recorded weight for the pair. The “impact score” for that pair was simply derived by subtracting the predicted SOC level using the lowest weight from the predicted SOC level when using the highest weight. We were then able to rank the importance of each “source”-“target” edge list pair for the prediction of SOC in our model both on a per-sample basis and also over all samples by assessing the mean and standard deviation “impact scores” across all of the samples.

### Reporting summary

Further information on research design is available in the Nature Research Reporting Summary linked to this article.

## Supplementary information

Supplementary Material

Reporting summary

Supplementary File 2

Supplementary File 1

## Data Availability

The experimental datasets used in this study are available from the ENA Sequence Read archive study PRJEB18701. The feature set that was used to train our best ML model is available in Supplementary File 2.
