# Supplementary material for: DGCNN approach links metagenome-derived taxon and functional information providing insight into global soil organic carbon
Source: NPJ Biofilms Microbiomes. 2024 Oct 26;10:113. doi: 10.1038/s41522-024-00583-9 (PMC11513995; doi:10.1038/s41522-024-00583-9)
Supplement: Supplementary file 1 — Supplementary Material [file 41522_2024_583_MOESM1_ESM.pdf]

Title: DGCNN approach links metagenome-derived taxon and functional information providing insight into global soil organic carbon

Authors:

Laura-Jayne Gardiner[1], Matthew Marshall[2], Katharina Reusch[1], Chris Dearden[2], Mark Birmingham[2], Anna Paola Carrieri[1], Edward O. Pyzer-Knapp[1], Ritesh Krishna[1], Andrew L. Neal[3]

Affiliations: [1] IBM Research Europe, Sci-Tech Daresbury, The Hartree Centre, Warrington, UK

[2] STFC Daresbury Laboratory, The Hartree Centre, Warrington, UK

[3] Net Zero and Resilient Farming, Rothamsted Research, North Wyke, EX20 2SB, UK

* Correspondence should be addressed to Laura-Jayne Gardiner and Andrew L. Neal

Laura-Jayne Gardiner: Laura-Jayne.Gardiner@ibm.com

Matthew Marshall: matthew.marshall@stfc.ac.uk

Katharina Reusch: Katharina@uk.ibm.com

Chris Dearden: chris.dearden@stfc.ac.uk

Mark Birmingham: mark.birmingham@stfc.ac.uk

Anna Paola Carrieri: acarrieri@uk.ibm.com

Edward O. Pyzer-Knapp: epyzerk3@uk.ibm.com

Ritesh Krishna: Ritesh.Krishna@uk.ibm.com

Andrew L. Neal: andy.neal@rothamsted.ac.uk

Supplementary Information

Supplementary Files

Supplementary File 1 – mapping statistics for the metagenomic soil samples
Supplementary File 2 – The feature set that was used to train our best ML model

Supplementary Notes

Supplementary Note 1. Bioinformatics analysis results for 189 soil metagenomes
Supplementary File 1 details the results of applying our ensemble of bioinformatic workflows to the Bahram et al. 189 global soil metagenomic samples. Increased read annotation was observed using a multi-software mapping approach. Supplementary Table 1 summarises the reads retained at each workflow stage across the samples. As observed previously (1), Kraken2's LCA approach classified the largest proportion of reads (90.3%). DIAMOND aligned fewer reads (84.1%) but assigned a larger proportion of reads to a species (57.1% versus 29.4% for Kraken2). HUMAnN3 aligned fewer reads (52.3%), potentially due to its reference database focus on functional annotation. Using multiple (intentionally redundant) approaches, on average across the samples, resulted in the collective assignment of 65.6% of quality-controlled reads to a species and 40.2% to a function (Supplementary File 1). This is an improvement over the best performance of a single workflow for annotating reads with species (DIAMOND 57.1%) and function (DIAMOND/Megan 34.5%). Although there is overlap between the reads each approach annotates, each added read annotations that were undetected by the other methods, providing an advantage over standard workflows where one software is typically selected for each processing step. This provides encouragement to explore software ensembles further. Furthermore, although not explored here, there is also potential to annotate additional reads by combining multiple databases for sequence mapping.

Bahram et al., found significant differences in the relative abundances of a range of phyla when considering the effect of habitat on these samples, they also noted a weak association between the taxonomic diversity of bacterial phyla and latitude, which was stronger and opposite in direction for fungi. Here, we assessed the microbial community at the species level, where similarly, most of our calculated alpha-diversity metrics revealed a statistically significant influence (ANOVA; $p<0.05$) of habitat (across eleven principal biomes), but also of longitude and/or latitude, on microbiome diversity

i.e., high-level environmental context (Supplementary Figure 1; Supplementary Table 2). There was also a consistent interaction between the habitat "Moist tropical forests" and geographical location longitude/latitude ($p < 0.05$). Supplementary Figure 2a-c shows that although sample sequencing coverage was not optimal, we detected differences in diversity based upon Hill numbers that may link to the habitat that sample was derived from.

We investigated between sample beta-diversity using rarefied species abundance counts (see Methods). Principal coordinates analysis (PCoA) explained around 50% of the variation in *Bray-Curtis* dissimilarity within the first two dimensions (Supplementary Figure 3). Additionally, habitat, latitude and longitude all yielded a statistically significant influence (PERMANOVA; $p<0.05$) upon microbiome diversity (Supplementary Table 3). Assessing beta-diversity using untransformed species counts and weighted *UniFrac* distances incorporating phylogenetic information (see Methods), explained a greater proportion of variability, around 62% within the first two dimensions (Supplementary Figure 4). Based on weighted *UniFrac* distances habitat, latitude and longitude still yielded a statistically significant influence (PERMANOVA; $p<0.05$) upon microbiome diversity (Supplementary Table 3), though discrete groupings based on habitats were unclear.

The five most abundant species across the sample set included some of the known most abundant soil bacteria such as Acidobacteria bacterium, Alphaproteobacteria bacterium, Verrucomicrobia bacterium, Actinobacteria bacterium and Chloroflexi bacterium. While the five most abundant functions across the sample set included K12132 (eukaryotic-like serine/threonine-protein kinase), K03088 (RNA polymerase sigma-70 factor), K01990 (ABC-2 type transport system ATP-binding protein), K03701 (exonuclease ABC subunit A) and K02004 (putative ABC transport system permease protein) involved in core pathways including metabolism, transcription, transport and nucleotide excision repair.

Supplementary Note 2. Initial classic ML testing and tuning. We trained and tested a series of ML models to compare the effect of data transformation techniques on SOC prediction for taxon-based and function-based abundance matrices separately (see Methods). For each feature set plus data transformation technique combination, we compared a range of regressors to predict the average SOC level for each location (depth 0-5 cm). We used the lowest mean absolute error (MAE) after cross validation (CV), balanced with the least overfitting between training and test data to select the "best" performing ML model (after fine tuning) for each combination of feature set and transformation technique. For all combinations, a Random Forest produced the best model. Furthermore, there was little difference between data transformation approaches. The best performing technique was normalisation separately for taxonomic and functional abundances according to the number of

mapped reads per sample ([counts per taxon or function/total number of reads mapped] * $1x10^6$) (Supplementary Figure 6). We used this approach in all subsequent analyses.

Using taxon abundance, the best performing ML model generated a MAE of 30.5 after CV, 18.5 on training and 24.8 on the held-out test data (Supplementary Figure 6a). For functional abundance the best ML model generated a MAE of 34.0 after CV, 18.3 on training and 25.4 on test data (Supplementary Figure 6b). Functional and taxonomic models performed similarly, showing some overfitting and the performance during CV being worse than on held-out test data meaning that performance might vary depending on the test set. The CV MAE equates to a ~8-9% error rate since the range of SOC measurements at depth 0-5 cm across our metagenomic samples was 8-392 g kg^{-1}.

We tested using alpha-diversity metrics in a ML model to predict SOC. The lowest observed MAE using alpha-diversity metrics was 39.4 after CV, 38.2 on training and 36.4 on the held-out test dataset (Supplementary Table 4) using a Gaussian Process. Very little variance across the test dataset was explained by the model ($r^2 < 0.2$) whether we used standard alpha-diversity metrics or Hill numbers. There was also a strong correlation between measured and predicted SOC levels using taxon and function features ($r = 0.79$-0.87) compared to a much lower correlation using alpha-diversity metrics ($r = 0.23$-0.45) (Supplementary Figure 7).

Supplementary Note 3. Environmental data profiling for soil microbiome collection. Parameters used to describe soil systems often exhibit high degrees of covariance. Therefore, we generated Pearson correlation coefficients between all environmental variables to determine the relationship between them (Supplementary Figure 9). Several climate or edaphic variables were correlated *e.g.*, rain vs total rain, rain vs soil water, air and soil temperature *etc*. Soil water had a stronger correlation to long-term rain than the daily rainfall on the day of sampling. Nitrogen, cations, and bulk carbon density were highly correlated as expected. Finally, bulk depth was correlated to SOC density and nitrogen. Highly correlated features were omitted from subsequent ML models to avoid multicollinearity. In this analysis our main aim was to predict SOC from the microbiome and in doing so to understand the dynamics of the microbiome in relation to global SOC levels across changing environments. As such, we eliminated those environmental variables that were highly correlated with SOC content, *e.g.*, organic nitrogen content, cation exchange capacity and bulk density of fine earth fraction of soil, since these might result in a ML model relying on these variables and masking subtler links between microbial taxa and SOC, providing no new insight into the dynamics of the system.

We combined our selected environmental variables with microbial function and species abundance counts. Using this feature set, the best ML model was generated using a Random Forest with a MAE

of 29.9 after CV with a high correlation observed between measured and predicted SOC levels for the test dataset (r = 0.82). The MAE after training was 16.6 (23.9 on test data) representing overfitting of our model.

We used feature selection (sequential univariate linear regression tests) to identify iteratively smaller sets of predictive features, each time re-training and testing the best ML model (Random Forest) to observe the effect of the reduced subset of features on model performance (see Methods). A subset of twenty-two features yielded the lowest observed MAE after CV of 29.2 (Fig.1a). This model still exhibited overfitting, but this was reduced from previous models with the MAE for the test dataset of 23.5 and 23.4 after training.

Supplementary Note 4. Environmental features predictive of SOC. Lower historical soil water content is predictive of higher SOC (Fig.2a), which contrasts with conventional thinking. However, the association is a weak negative correlation (r = -0.053, p = 0.49). Additionally, the ERA5-derived soil water content is itself a prediction based upon several measurements, including soil texture (or classification), soil depth, and the underlying groundwater level. Comparing low (0-0.3 $m^3\ m^{-3}$) and high (0.3-0.5 $m^3\ m^{-3}$) soil water contents, identifies a statistically significant correlation with SOC content that is positive for lower soil water (r = 0.19, p = 0.05) but negative for higher soil water contents (r = -0.24, p = 0.014). Since the soil water content is linked to groundwater level, then this pattern supports the observation by (2) of SOC content increasing with groundwater depth up to a point then decreasing at higher depths.

Supplementary Note 5. Analysis of Spiec-easi networks. We observed generally a greater degree of clustering in the networks representing the Hyphomicrobiales compared to *Ca*. Gallionella and *Astrumicrobium* networks (global clustering coefficient: *Ca*. Gallionella 0.24, *Astrumicrobium* 0.22, *Microvirga thermotolerans* 0.19, *Mesorhizobium loti* 0.25, *Mesorhizobium kowahii* 0.27, *Tardiphaga* sp. vice274 0.28, *Microvirga lupini* 0.3). The average weighted degrees of the Hyphomicrobiales networks were similar to that of *Ca*. Gallionella (range 0.97 - 1.1) but the respective graph densities were much lower (range 0.03 - 0.05). The mycorrhizal fungus *Russula emetica* was present in the *Mesorhizobium kowahii* association network. Other fungi were associated with the networks of *Tardiphaga* sp. vice274 (*Umbelopsis inacea*) and *Microvirga lupini* (the endolichenic *Daldinia childiae* and the ascomycete *Fusarium albosuccineum*).

Supplementary Note 6. Sample-specific local investigations of DGCNN predictions for two samples. For S234 the DGCNN predicted a SOC content of 177 g kg^{-1} (measured value 193 g kg^{-1}) which was a large improvement on the previous prediction of 124 from the (non-graph) SVM i.e.,

predicted value closer to the measured value. For S72 that had a measured SOC level of 49 g kg^{-1} the DGCNN also improved the SVM prediction of 62.9 to 57.9 g kg^{-1}. For these two samples we used our permutation importance approach to assess the impact of node pairs for SOC predictions (Supplementary Figure 14a-b). Prediction of SOC for sample S234 (high carbon) was sensitive to both positive and negative impact node pairs, the majority of them associated with Verrucomicrobiaceae. These *Astrumicrobium roseum* LW23 ‖ function node pairs are shown in Supplementary Figure 14a i.e., red bars for positive impact scores indicating that a high edge weight (high coverage) for the node pair supports an increased prediction of SOC. There is also a red bar corresponding to *Oxytricha granulifera* ‖ K00782 (*L*-lactate dehydrogenase complex protein, *lldG*, associated with carbohydrate metabolism). In contrast there are two relatively large blue bars corresponding to *Verrucomicrobium* sp. GAS474 ‖ K00879 (*L*-fuculokinase, EC:2.7.1.51, *fucK*, associated with fructose and mannose metabolism and pentose and glucuronate interconversions), K01740 (*O*-acetylhomoserine (thiol)-lyase, EC:2.5.1.49, *metY*, associated with cysteine and methionine metabolism), K03452 (magnesium/proton exchanger, MHX) and K03603 (GntR family transcriptional regulator, negative regulator for fad regulon and positive regulator of *fabA*, *fabR* associated with genetic information processing) and secondly, paired with K23047 (E3 ubiquitin-protein ligase UNKL EC:2.3.2.27, associated with genetic information processing). These bars, plus two blue *Verrucomicrobium* sp. GAS474-related bars, give clear examples of when different functions from the same species can be unequally predictive for a given sample. The blue bars represent negative scores which indicate that a low weight (low coverage) for the node pair in the graph drives an increased prediction of SOC level in this sample.

By comparison, S72, a lower carbon sample (Supplementary Figure 14b), had only positive impact node pairs affecting its prediction. Again, a large number of *Astrumicrobium roseum* LW23 ‖ function node pairs encompassed the 3 red bars in Supplementary Figure 14b. The four functions in the most impactful group (K01588, 5-(carboxyamino)imidazole ribonucleotide mutase, EC:5.4.99.18, *purE* associated with *de novo* purine biosynthesis; K01615, glutaconyl-CoA decarboxylase subunit alpha, EC:7.2.4.5, *gcdA* associated with butanoate metabolism and benzoate degradation; K03406, a methyl-accepting chemotaxis protein, *mcp* associated with bacterial chemotaxis; and K06066-like function, were not in the most (positive) impactful group for S234 (shown as 159 functions in Supplementary Figure 14a). This comparative analysis suggests what might be important for an individual prediction, but we do not explicitly assess the sample-specific edge weight of the node pair, only the impact of it being a high or low prediction (see Methods). Therefore, we have not considered why S234 might have a high predicted SOC level and S72 a low level e.g., what do these samples actually show? S234 shows significant presence of *Astrumicrobium roseum* LW23 (159 functions with

coverage) which has a positive influence on higher SOC level prediction when present. S234 conversely shows no coverage of any of the four top negative functions (largest blue bar in Supplementary Figure 14a) associated with species *Verrucomicrobium* sp. GAS474*,* then very low coverage of only eight other functions associated with this organism that all have a positive influence on increasing SOC when low*.* This may explain its predicted high level of SOC. In contrast, S72 shows presence of *Astrumicrobium roseum* LW23 but at a lower level compared to S234 (e.g., coverage spanning mainly the four functions that form its most predictive group of node pairs) which may explain its lower-level prediction.

Supplementary Figures

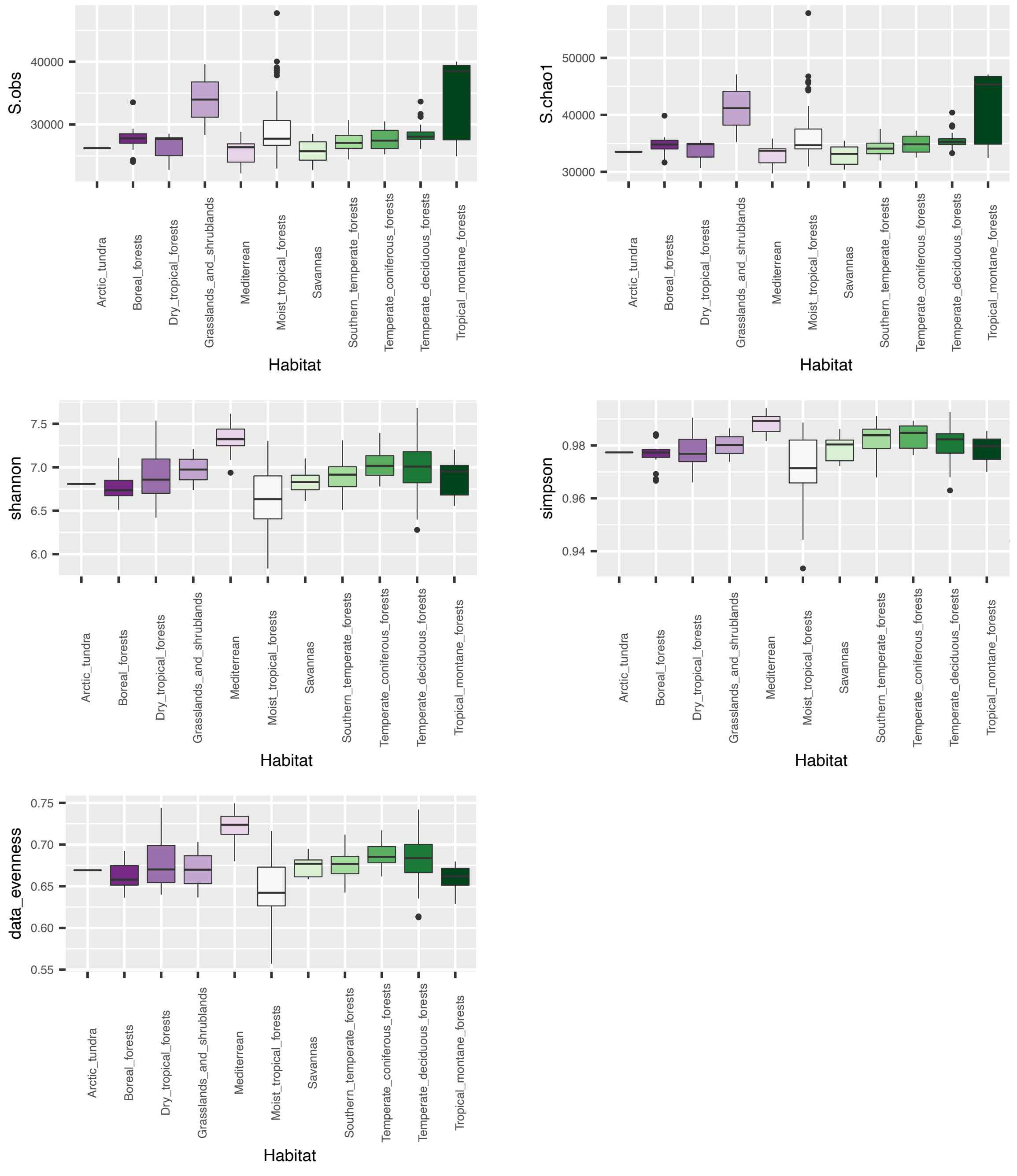

Supplementary Figure 1. Comparing alpha diversity metrics across different habitats. Box plots showing variation across 11 main habitats as defined by Bahram et al, for each of the diversity metrics; species richness (S.obs), Chao-1 (S.chao1), Shannon index (shannon), Simpson index (simpson) and Pielou's evenness (data_evenness). We investigated the alpha-diversity metrics as calculated from raw counts (see Methods). It appears that intra-variability is lower than inter-variability among the different habitats or locations.

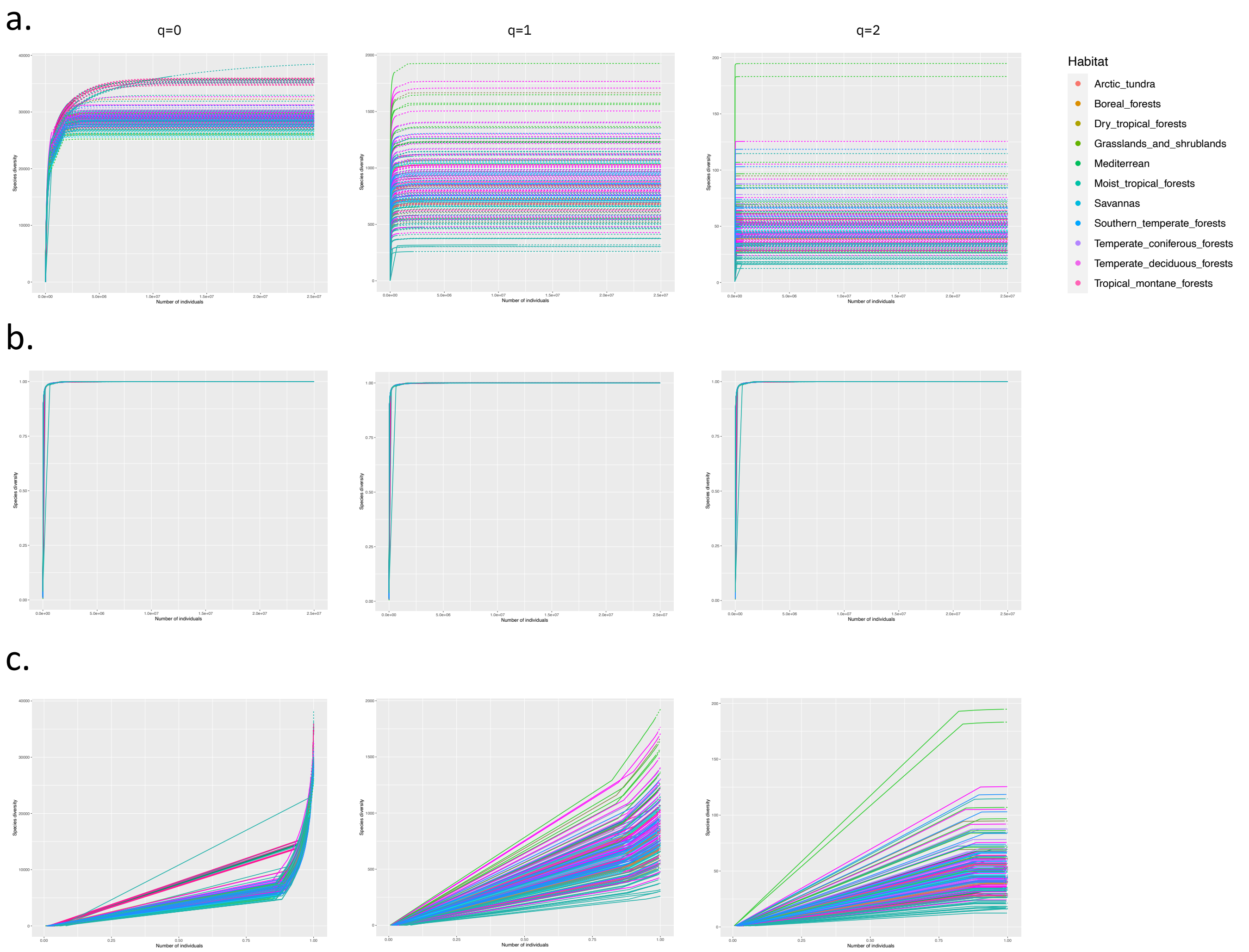

Supplementary Figure 2. Comparing alpha diversity metrics across different habitats using Hill numbers. We explored diversity of bacterial, archaea and fungi communities separately, this time computed as Hill numbers for species richness, Shannon diversity and Simpson diversity (see Methods). Here, we show the results of this analysis for the bacterial community, since near-identical trends were observed across archaea and fungal communities. Hill numbers of order q: species richness (q=0), Shannon diversity (q=1, the exponential of Shannon entropy) and Simpson diversity (q=2, the inverse of Simpson concentration). Lines are colour coded as per the 11 main habitats as defined by Bahram et al (3), and detailed in the legend in the top right corner. Plots correspond to (a) sample-size-based R/E sampling curves (b) sample completeness curves to show how the sample coverage estimate varies as a function of sample size, and (c) coverage-based R/E sampling curves of diversity estimates for raw abundance counts and extrapolated samples with sample completeness (as measured by sample coverage) up to the coverage value of 25 million reads (our largest sample size).

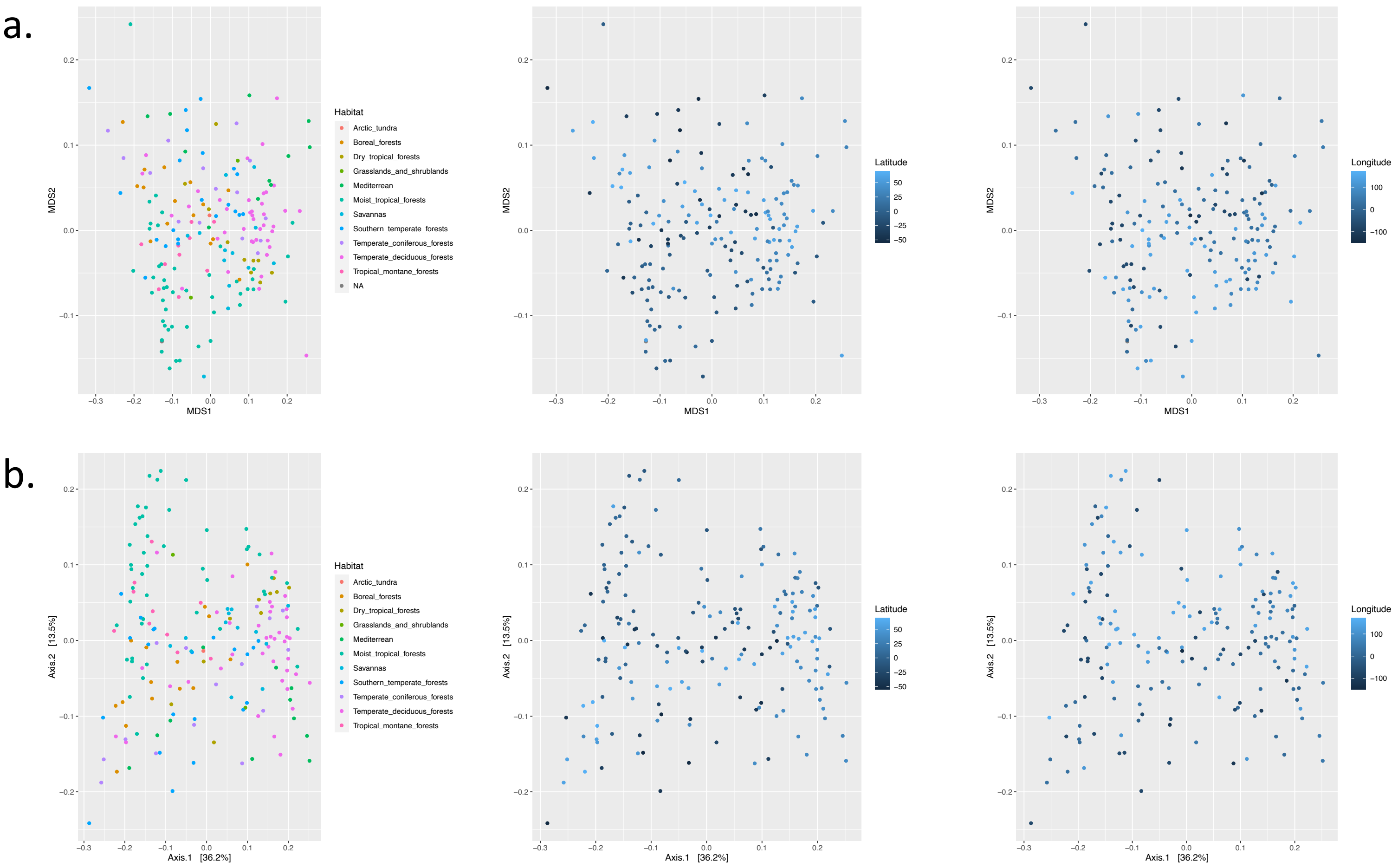

Supplementary Figure 3. Comparing Principal coordinates analysis (PCoA) and Non-metric Multidimensional Scaling (NMDS) plots of beta diversity metrics. (a) NMDA and (b) PCoA were used to represent the pairwise dissimilarity between metagenomic soil samples in a 2-dimensional space using the Bray-Curtis dissimilarity index. Scatter plots from left to right represent the colour coding of samples based on their habitat of origin, latitude and longitude respectively.

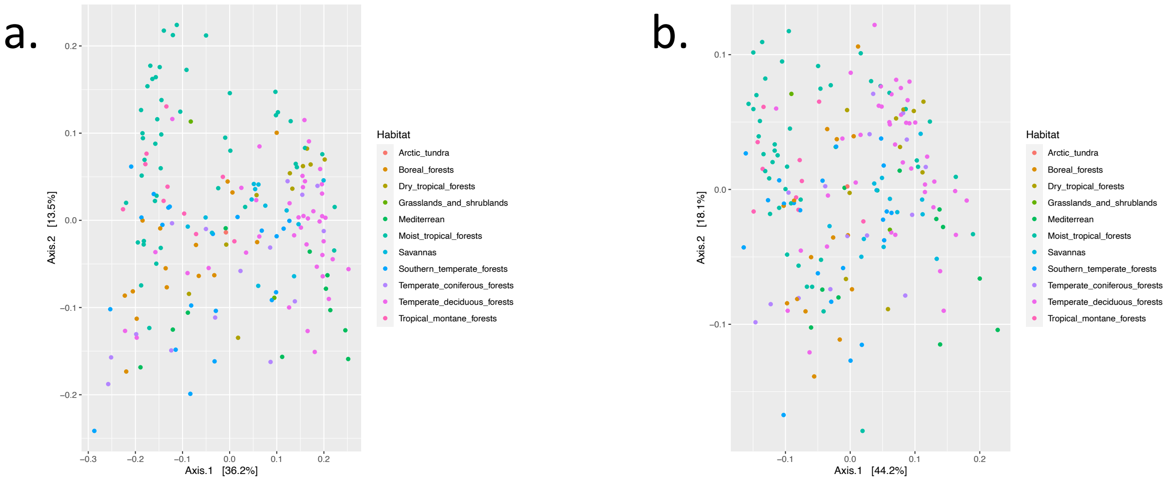

Supplementary Figure 4. Comparing Principal coordinates analysis (PCoA) plots of beta diversity metrics. PCoA was used to represent the pairwise dissimilarity between metagenomic soil samples in a 2-dimensional space using the **(a)** *Bray-Curtis* dissimilarity index and **(b)** weighted *unifrac* distances. Scatter plots represent the colour coding of samples based on their habitat of origin.

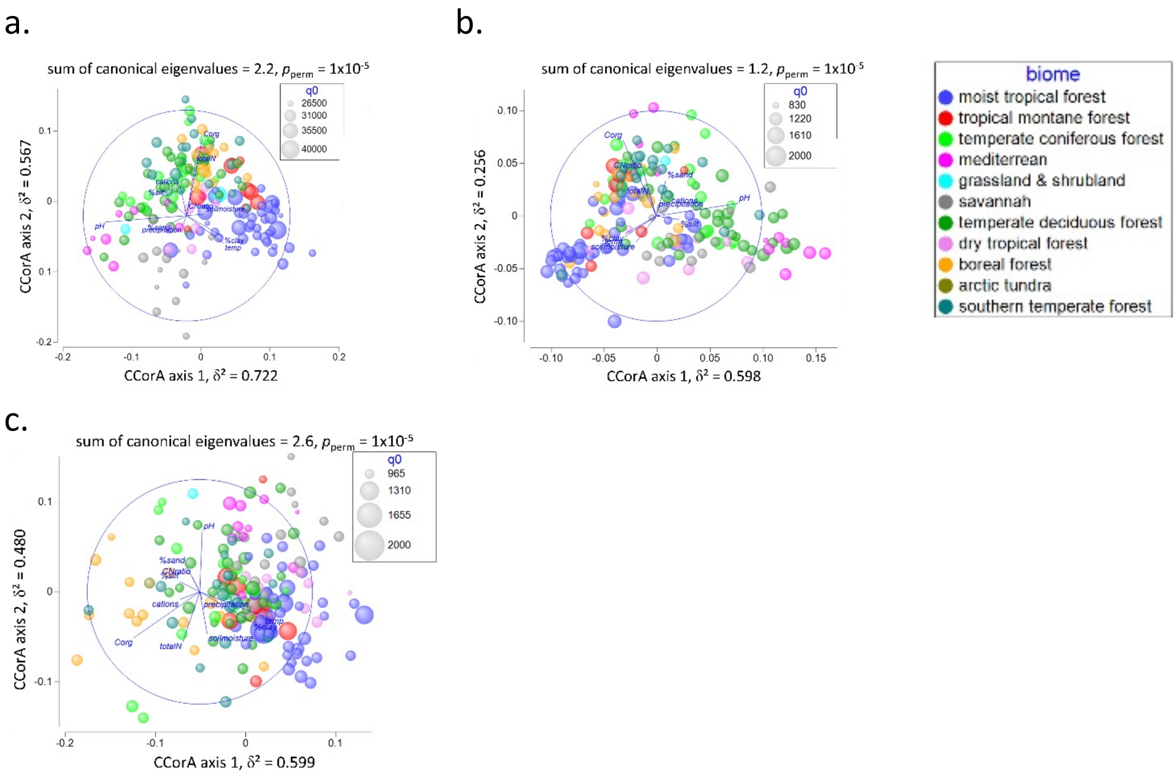

Supplementary Figure 5. Canonical correlation analysis (CCorA). To investigate general relationships between climate and edaphic variables and the phylogenetic distinctness of soil microbial communities, we used canonical correlation analysis (CCorA) to identify linear combinations of environmental variables and linear combinations of phylogenetic distance between soil microbial communities that were maximally correlated with one another, using weighted UniFrac as the measure of phylogenetic distance. **Corg** corresponds to SOC level reported elsewhere in this study, **totalN** corresponds to the total Nitrogen in the soil (g kg^{-1}), **C/N ratio** corresponds to the carbon to nitrogen ratio, **%sand or %silt or %clay** correspond to the volumetric fraction of sand/silt/clay-proportion of sand/silt/clay particles respectively in the fine earth fraction (g $100g^{-1}$ (%)), **cations** represent the cation exchange capacity of the soil (cmol(c) kg^{-1}), **pH** is the soil pH, **precipitation** represents the total rainfall for the specific day the sample was taken (m) and **temp** corresponds to the temperature on the day the microbiome sample was taken (at lunchtime) (degrees Celsius). Results of the analysis are depicted for **(a)** bacterial **(b)** archaeal and **(c)** fungal communities. *p* determined from 99,999 permutations in each case.

a.

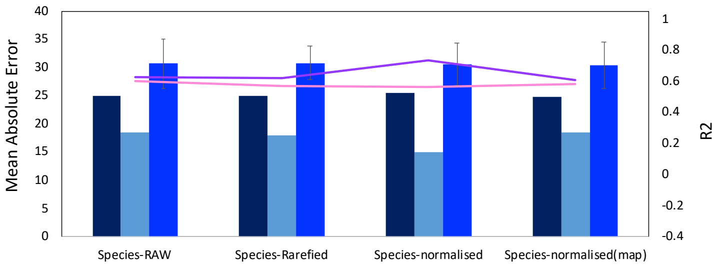

	Species abundances RAW	Species abundances Rarefied	Species abundances normalised	Species abundances normalised (map)
Test r2	0.599	0.571	0.566	0.58
Test MAE	24.988	24.959	25.54	24.773
Test explained variance	0.61	0.595	0.581	0.601
Train MAE	18.43	17.983	14.953	18.538
Mean MAE CV	30.703	30.856	30.619	30.454
SD MAE CV	4.427	2.961	3.743	4.175

b.

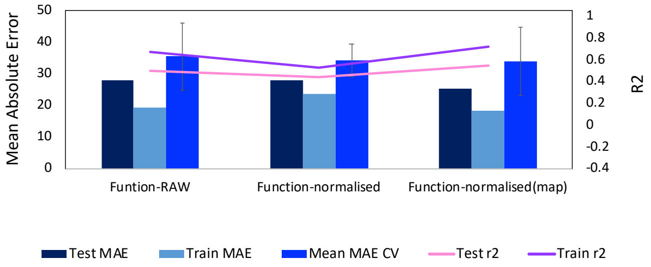

	Function counts RAW	Function counts normalised	Function counts normalised (map)
Test r2	0.498	0.442	0.549
Test MAE	27.96	27.77	25.402
Test explained variance	0.501	0.492	0.561
Train MAE	19.329	23.654	18.327
Mean MAE CV	35.441	34.113	33.973
SD MAE CV	5.168	4.07	3.415

Supplementary Figure 6. Comparing ML models for taxonomic and functional abundance matrices using different transformation techniques. Plots to the left show ML model performance for a range of metrics (see legend at bottom of figure) including Mean Absolute Error (MAE) on the training dataset (Train MAE), MAE on the held-out test set (Test MAE), the mean MAE value after five-fold cross-validation (Mean MAE CV), and coefficient of determination (r^2) values for the training and held-out test dataset (Train r^2 and Test r^2). Plots for **(a)** taxonomic (species) abundances and **(b)** functional abundances (KEGG) compare raw abundance counts (Species/Function-RAW) with transformation techniques such as rarefication (Species-Rarefied), normalisation on the number of mapped and unmapped reads per sample after quality control (Species/Function-normalised) and normalisation based only on the number of mapped reads per sample (Species/Function-normalised(map)) (see Methods). The tables to the right numerically summarise the same data shown in the plots.

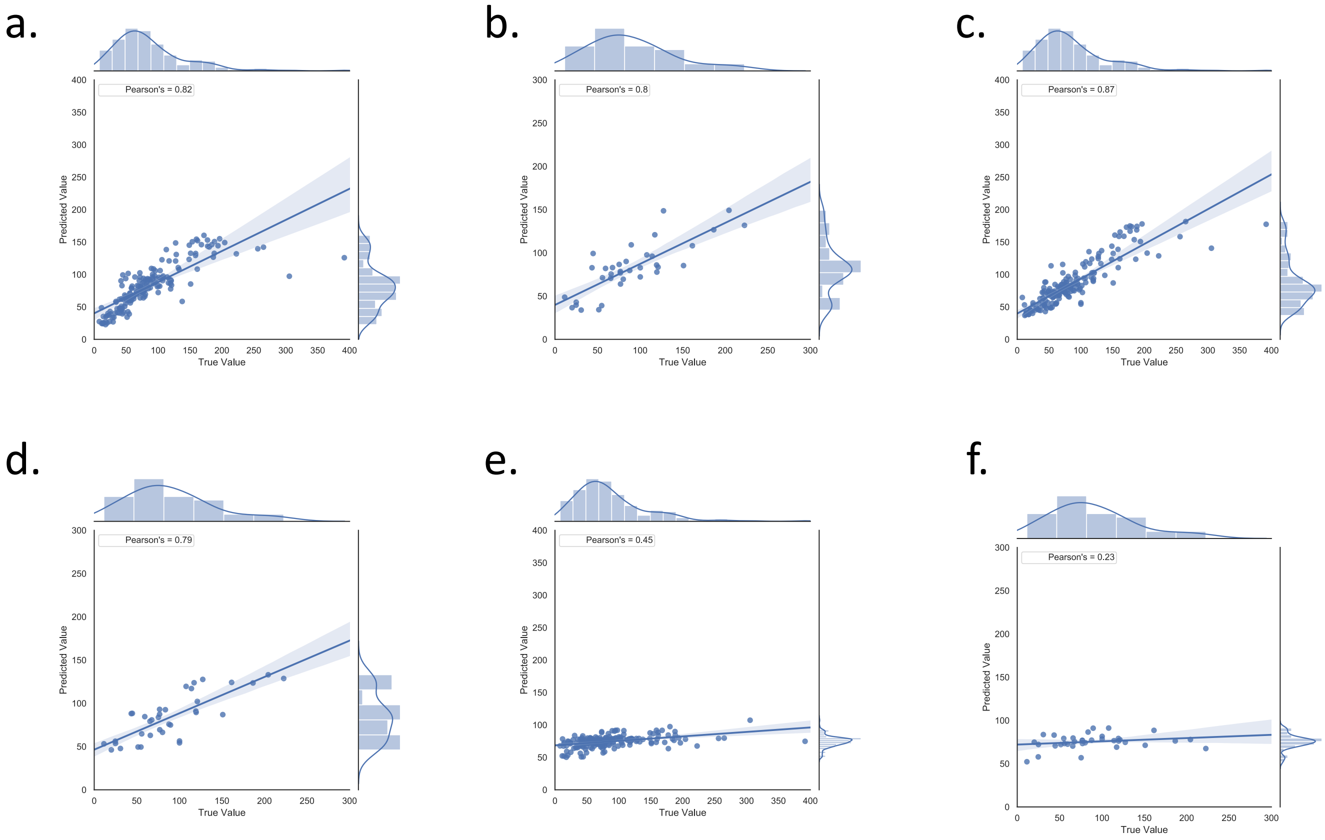

Supplementary Figure 7. Comparing taxonomic, functional and alpha diversity derived feature sets for ML. Plots show the true versus predicted values for soil organic carbon levels (dg/kg) for each of the locations associated with the soil metagenomic samples. The Pearson's correlation co-efficient is also shown for each plot. Plots correspond to predictive models generated with a range of different feature sets **(a)** taxonomic (species) abundances for all samples (r= 0.82, p< 0.00001), **(b)** taxonomic (species) abundances for only the held-out test samples (r= 0.80, p< 0.00001), **(c)** functional abundances for all samples (r= 0.87, p< 0.00001), **(d)** functional abundances for only the held-out test samples (r= 0.79, p< 0.00001), **(e)** alpha diversity metrics for all samples (r= 0.45, p< 0.00001) and **(f)** alpha diversity metrics for only the held-out test samples (r= 0.23, p=0.177).

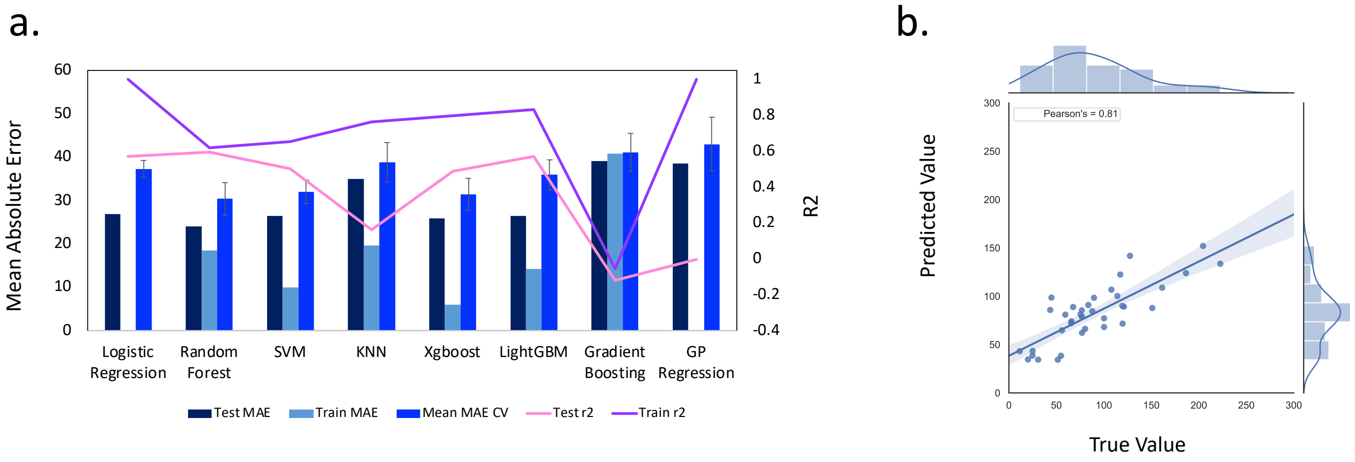

	Logistic Regression	Random Forest	SVM	KNN	Xgboost	LightGBM	Gradient Boosting	GP Regression
Test r2	0.57	0.594	0.502	0.159	0.487	0.568	-0.121	-0.005
Test MAE	26.833	23.93	26.352	34.842	25.868	26.376	38.962	38.452
Test explained variance	0.599	0.616	0.529	0.281	0.515	0.571	0	0
Train r2	1	0.616	0.652	0.763	0.795	0.831	-0.055	1
Train MAE	0	18.441	9.879	19.616	5.96	14.146	40.748	0
Train explained variance	1	0.62	0.673	0.767	0.803	0.831	0	1
Mean MAE CV	37.234	30.29	31.932	38.788	31.3	35.835	41.062	42.916
SD MAE CV	1.947	3.76	2.742	4.565	3.703	3.499	4.41	6.201

Supplementary Figure 8. Comparing ML models for combined taxonomic abundances, functional abundances, and alpha diversity metrics. Plots for **(a)** show ML model performance for a range of regressors and metrics- as described in Supplementary Figure 6 (see legend at bottom of figure). ML models are trained using the combined features for normalised taxonomic abundance, normalised functional abundance and alpha diversity metrics to predict SOC levels. The table below numerically summarises the same data shown in the plot. **(b)** For the same feature set in (a), this plot shows the true versus predicted values for SOC content (g kg^{-1}) for each of the soil metagenomic samples from the held-out test dataset. The Pearson's correlation coefficient (*r*) is also shown (0.81), this correlation was statistically significant (*p*< 0.00001).

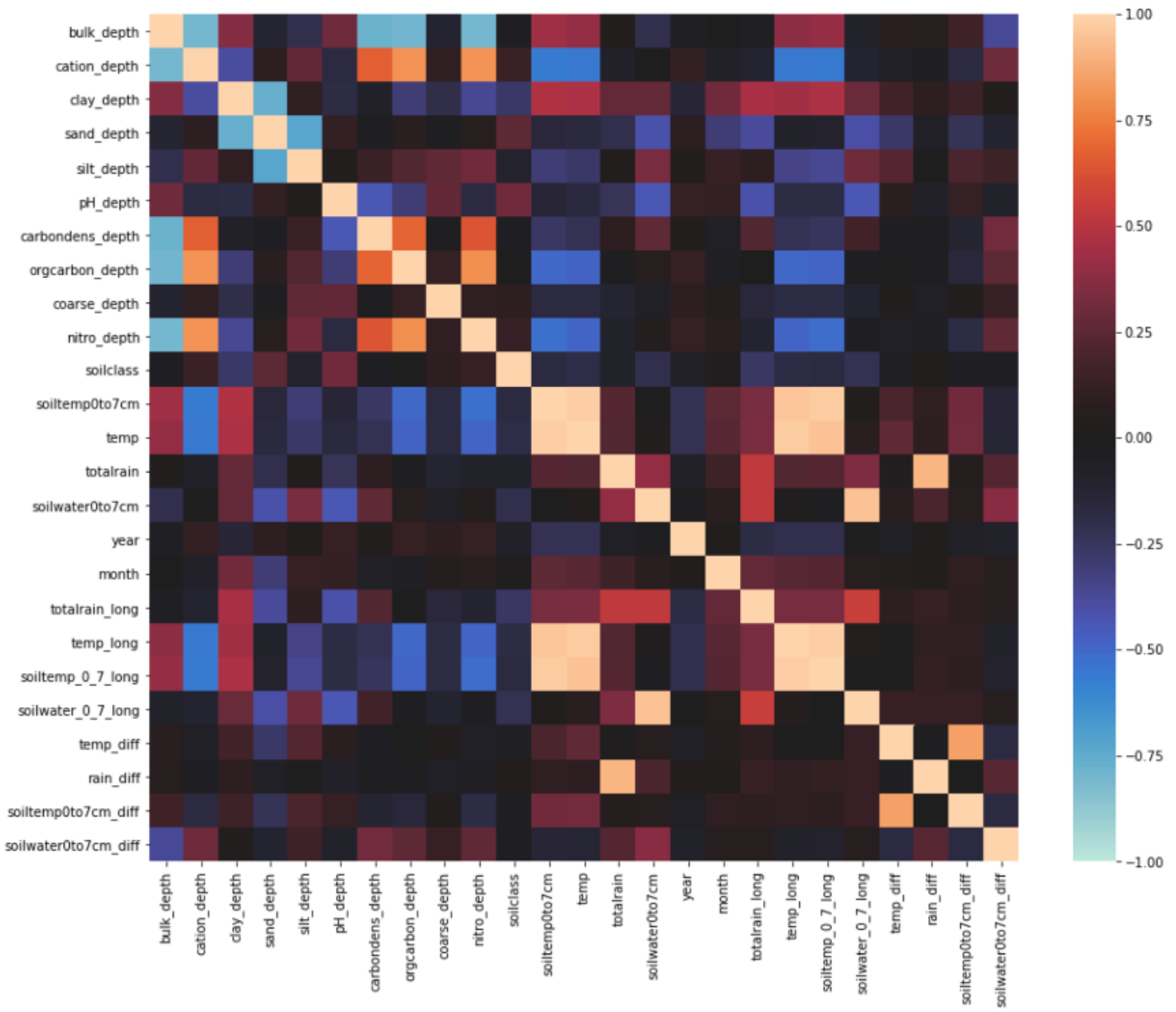

Supplementary Figure 9. Correlation matrix heatmap for environmental variables matching metagenomic soil sample geo-locations. Pearson correlation to understand the relationship between features. See Table S5 for abbreviations, and otherwise, **bulk_depth** refers to the bulk density of the fine earth fraction ($kg\ dm^{-3}$), carbondens_depth refers to the organic carbon density in kg/m^3 (here SOC is represented as orgcarbon_depth) , **year** refers to the year of sampling, **month** refers to the month of sampling, totalrain_long/soiltemp_0_7_long/soilwater_0_7_long all refer to the respective measurement i.e., totalrain/soiltemp0to7cm/soilwater0to7cm, when a 20-year average is taken for a locale.

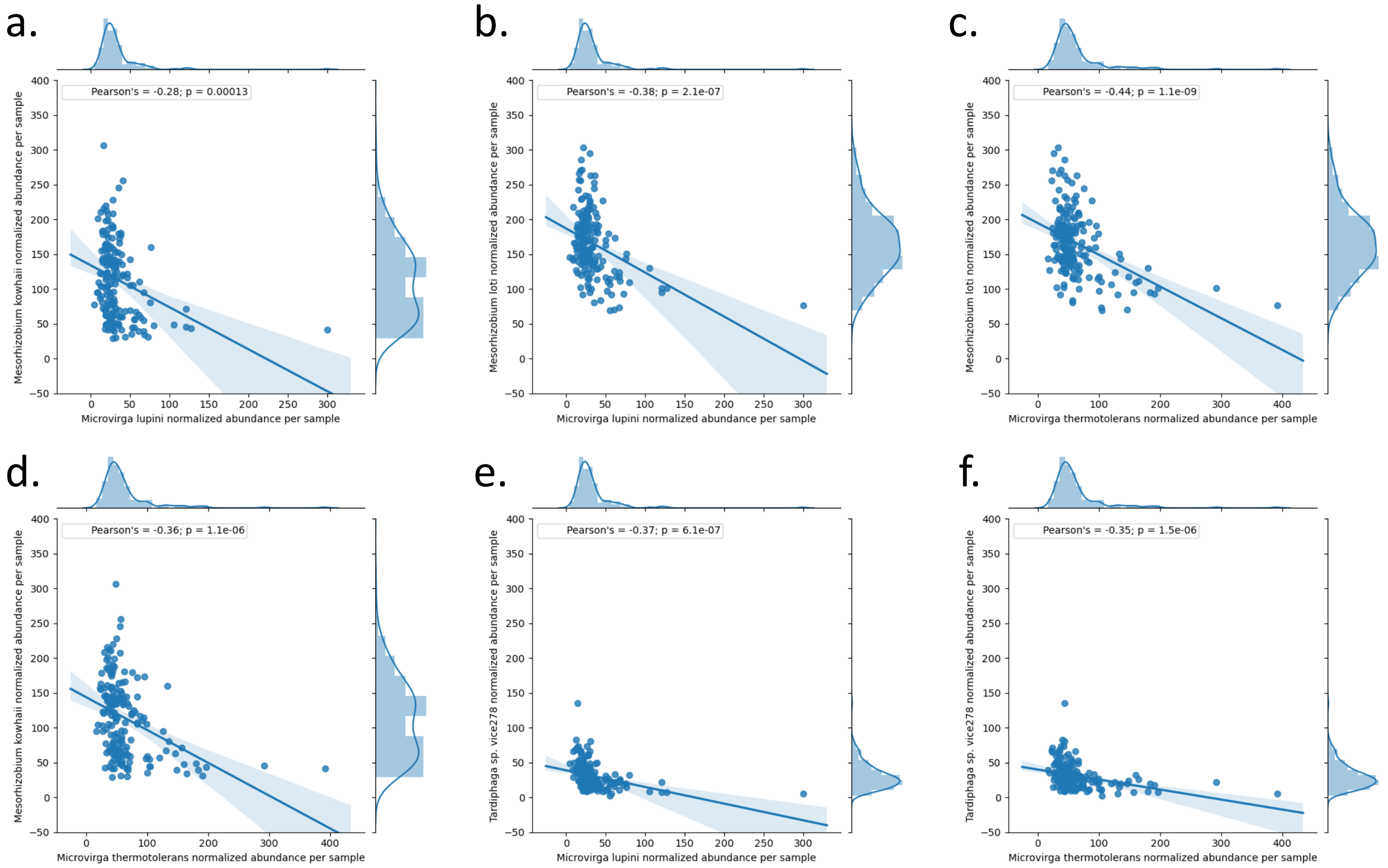

Supplementary Figure 10. Comparing the correlations per sample between normalized species abundances. Plots show the normalized species abundances associated with the soil metagenomic samples (individual species in plots **(a-f)** being compared are as labelled on the x and y axes). Here we focus on the trends between groups of diazotrophs that are in the twenty-two most predictive features in Table.1 and show opposite (positive/negative) associations with SOC content. The Pearson's correlation co-efficient is also shown for each plot, and associated *p*-values are shown alongside these values.

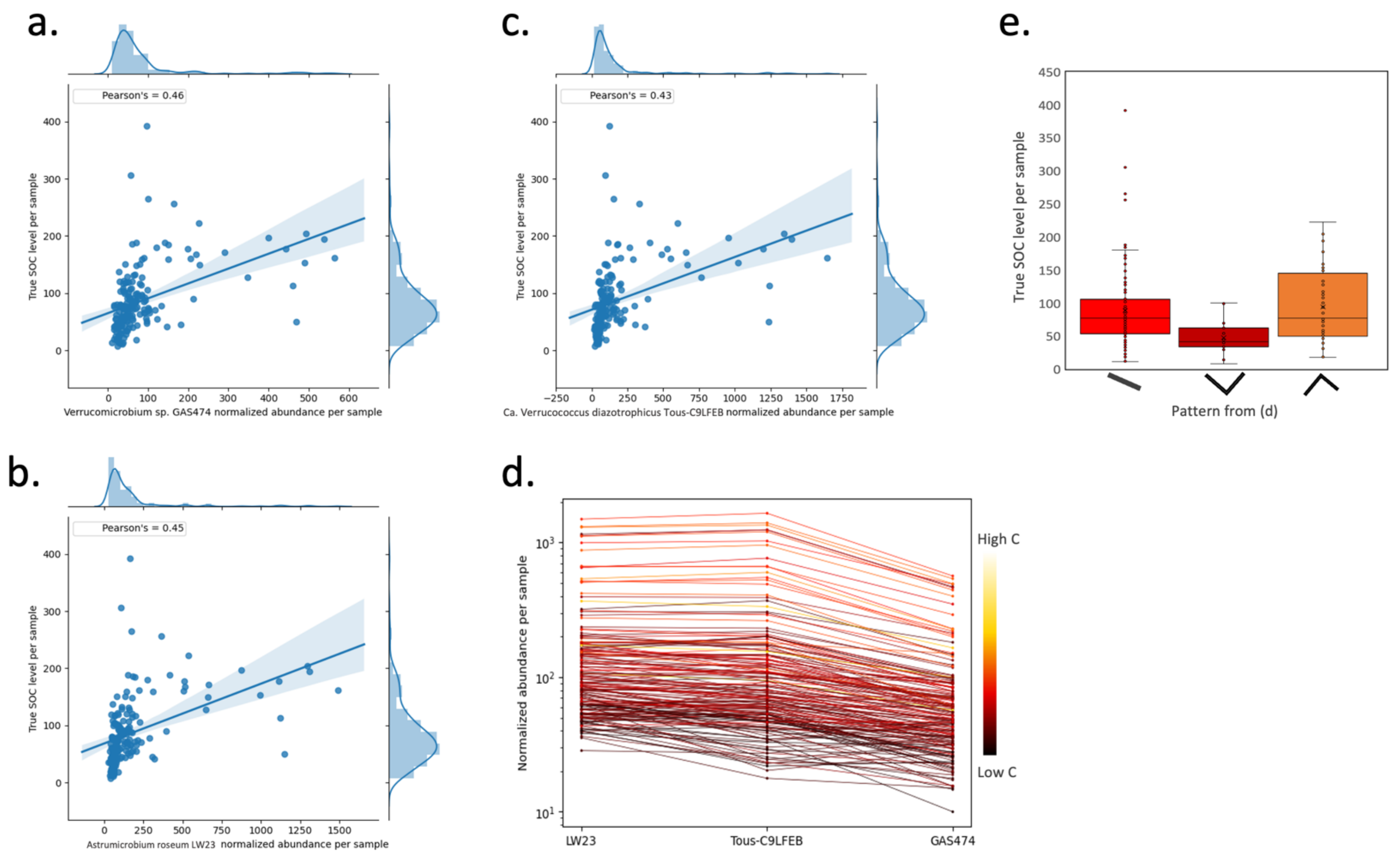

Supplementary Figure 11. Investigating the Verrucomicrobiacaea in the top twenty-two most predictive features from classic ML. Plots **(a-c)** show correlations between normalized species abundance per sample (species labelled on *x*-axis) with SOC content (dg kg^{-1}) (*y*-axis) of each soil metagenomic sample location. The Pearson's correlation co-efficient is shown for each plot (all associated *p*-values are <0.00001). Line plot **(d)** visualizes species abundance per sample after normalization (*y*-axis) and its variation between the three Verrucomicrobiaceae i.e., relative species abundance; A*strumicrobia roseum* LW23 (labelled LW23), Ca. Verrucococcus diazotrophicus Tous-C9LFEB (labelled Tous-C9LFEB) and *Verrucomicrobium* sp. GAS474 (labelled GAS474). Line colours describe the true SOC content of the sample as per the colour bar to the right of the plot. Box plot **(e)** depicts the true SOC content per sample (*y* axis) where the three boxes group samples according to their observed patterns from (d). The three observed patterns include "\" [abundance of LW23 highest, then Tous-C9LFEB lower, then GAS474 lowest], "\/" [abundance of LW23 and GAS474 highest, then Tous-C9LFEB lowest] and "/\" [abundance of Tous-C9LFEB highest, then LW23 and GAS474 lowest]. Comparing box plots using a two-tailed unpaired t test, samples with "\" had significantly higher SOC contents than "\/" (*t*=2.6, df=125, *p*=0.0096) and samples with "\/" had significantly lower SOC contents than "/\" (*t*=3.2, df=61, *p*=0.0025).

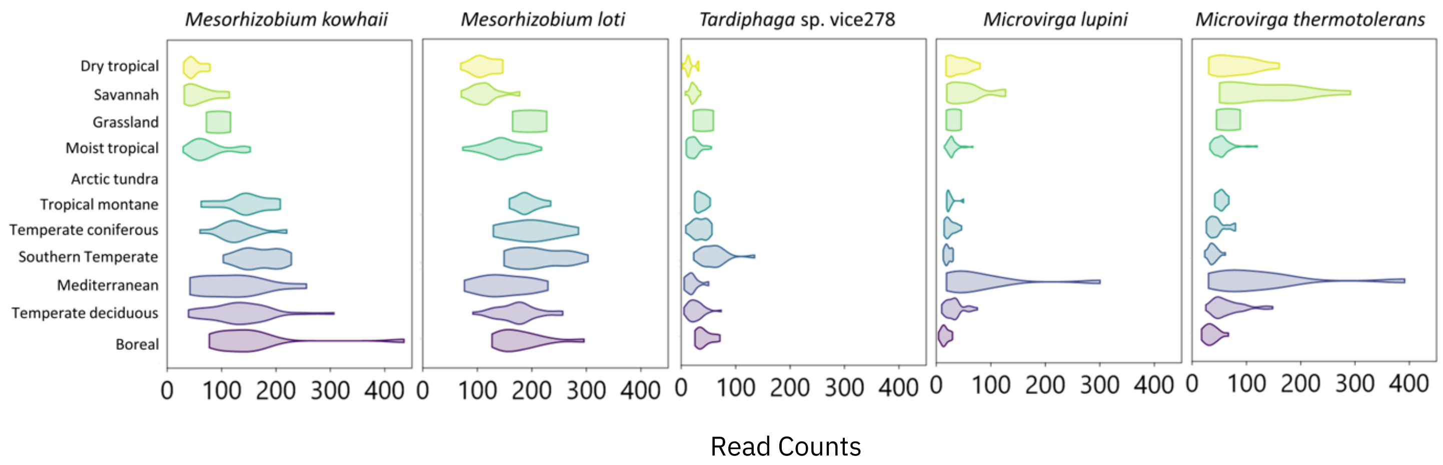

Supplementary Figure 12. Violin plots to compare key predictive species abundances across habitats. Here we show violin plots for our second most predictive feature *Tardiphaga* sp. vice278, nitrogen fixing diazotrophs positively associating with SOC *Mesorhizobium kowhaii* and *Mesorhizobium loti* compared to those negatively associating with SOC *Microvirga thermotolerans* and *Microvirga lupini*. Read counts correspond to normalized abundances per species.

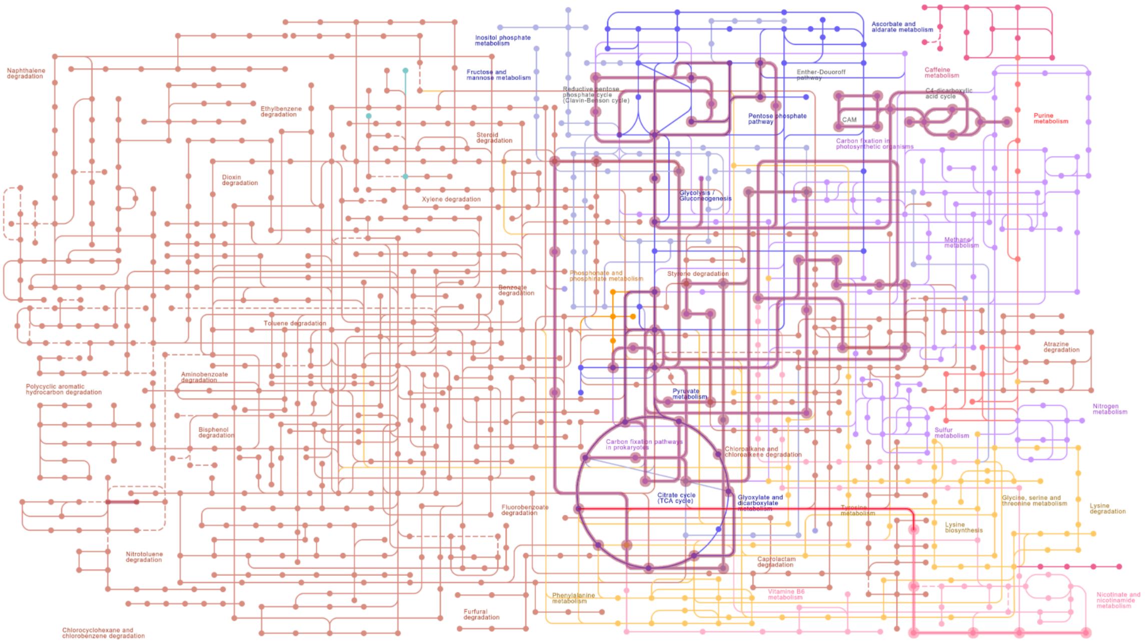

Supplementary Figure 13. KEGG reference pathway "Microbial metabolism in diverse environments". This reference pathway comes from KEGG ((4)). We have used the functionality of the website to highlight the carbon fixation metabolic pathway in dark pink. The pathway for Nicotinate degradation has also been highlighted in light pink, while the specific KEGG function K01799 that is part of this nicotinate degradation pathway is coloured in red.

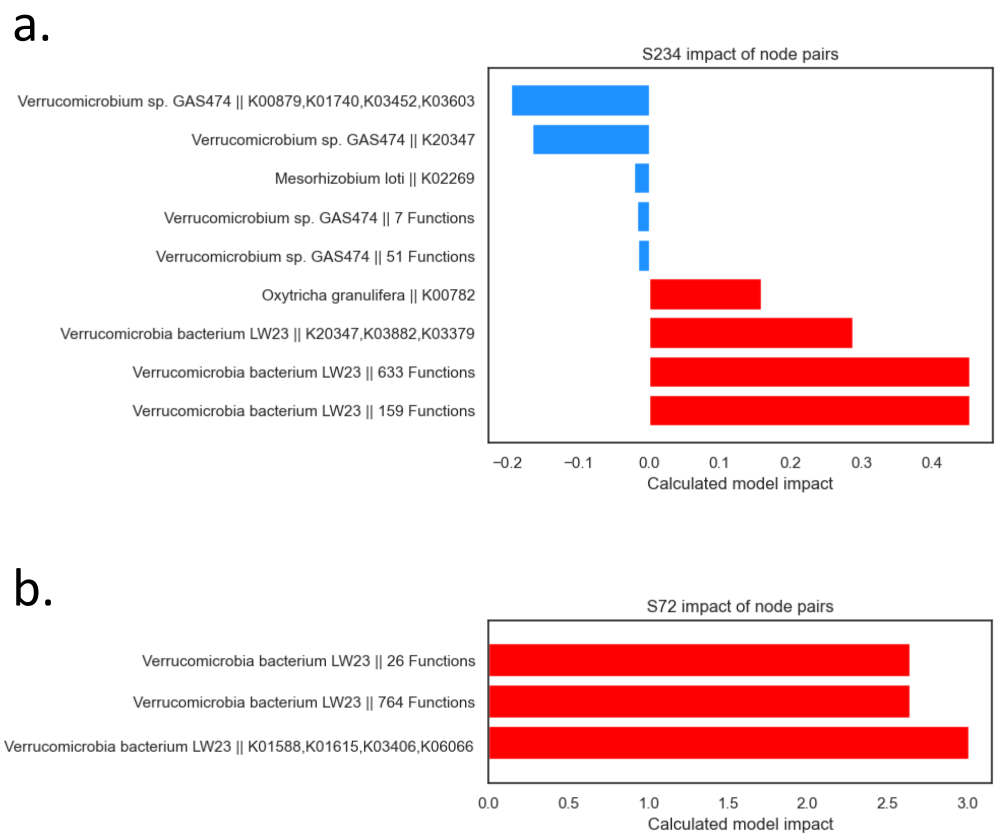

Supplementary Figure 14. Local interpretation of our DGCNN predictions for Samples S234 and S27. Bar plots for **(a)** sample S234 and **(b)** sample S72, where we report the calculated "impact" of any taxon ‖ function node pairs where the impact value was greater than zero (see Methods for description of calculation). The length of the bar corresponds to the impact value of the node pair. Multiple node pairs that have the same impact value are combined into groups and shown as one representative bar that is the length of one of the node pairs impacts (as described in the labels on the *y*-axis). Bars are coloured blue if the impact score is negative and red if the score is positive. A positive score indicates that a high weight (high depth of sequencing coverage) for the node pair in the graph drives an increased prediction of SOC level by the calculated impact value. A negative score indicates that a low weight (low depth of sequencing coverage) for the node pair in the graph drives an increased prediction of SOC level by the calculated impact value.

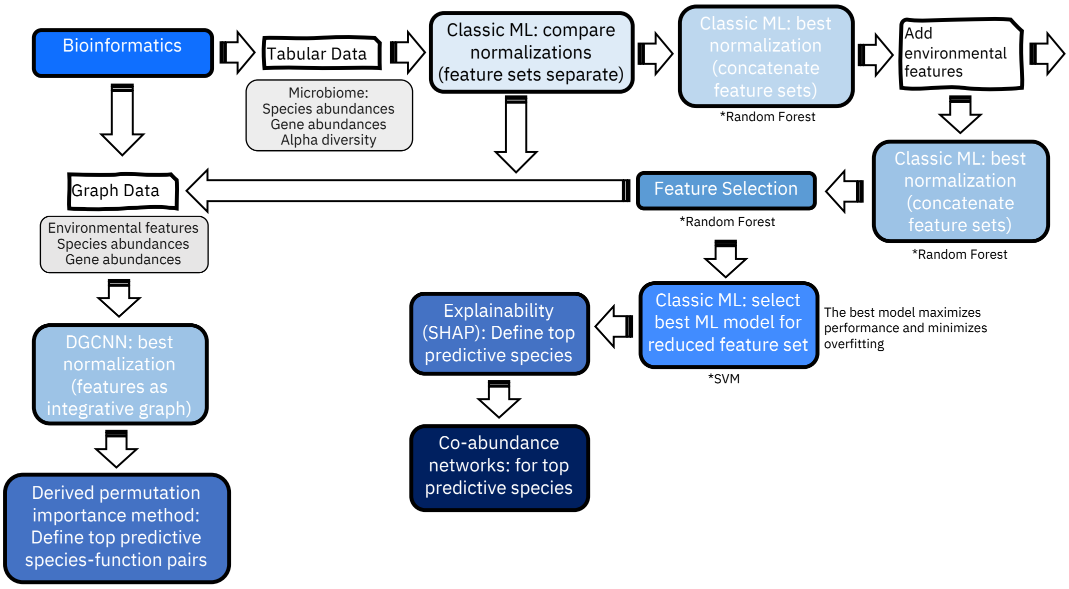

Supplementary Figure 15. Flow diagram of high-level methodology overview for prediction of SOC levels. Throughout the manuscript we follow a process that includes bioinformatics, normalisation of features, feature integration (via graphs and tabular concatenation), classic ML, feature selection, DL, model explainability/interpretability and generating co-abundance networks for predictive features. All ML/DL tasks refer to the prediction of SOC level. Both the classic and graph ML workflows require normalised, per-processed input data and splitting of the input dataset into train and test sets that are conserved across all analyses. The classic ML workflow involves training and testing a series of ML regressors, each time performing model optimisation comparing a range of hyper parameters. This is followed by a process to select the best performing model per feature set. The DGCNN refers to a single model architecture. See methods for more details.

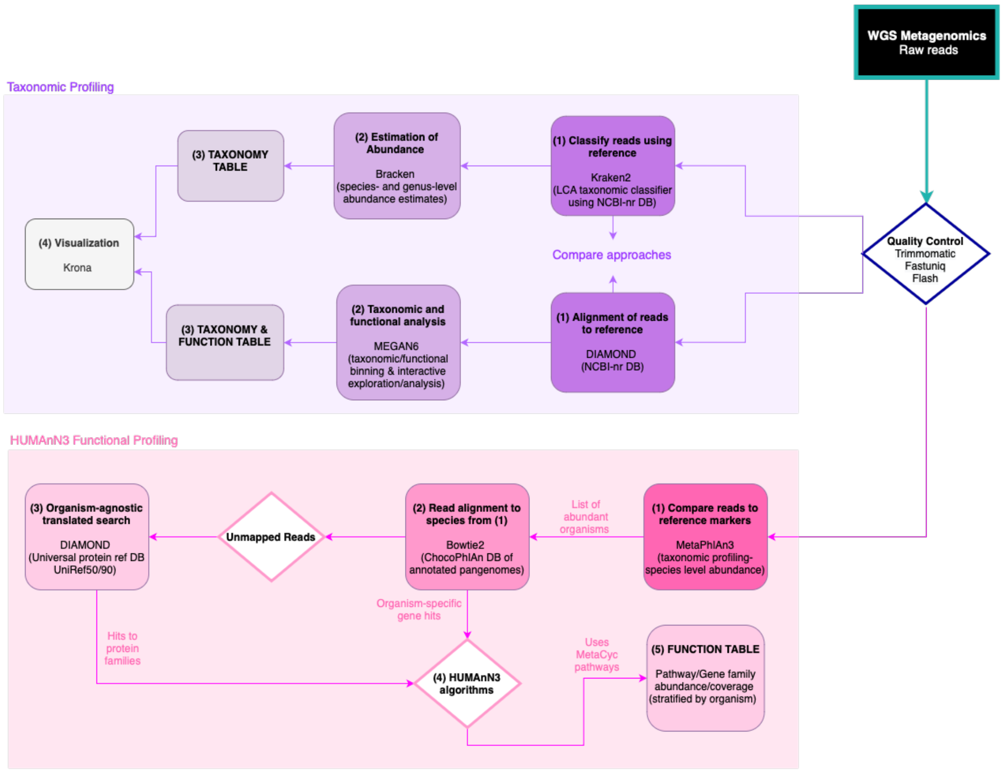

Supplementary Figure 16. Flow diagram of purpose built metagenomic bioinformatic workflow for taxonomic and functional annotation. Detailing the two complementary strands of the workflow which mainly focused on **(purple)** taxonomic profiling - with additional functional annotation possible and **(pink)** functional profiling.

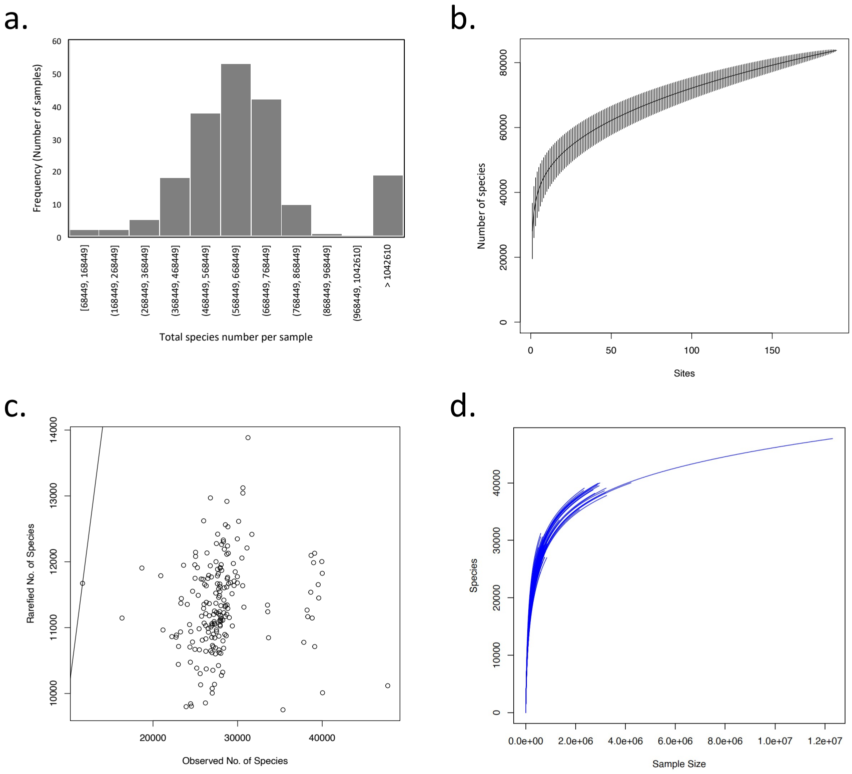

Supplementary Figure 17. Assessing taxonomic species diversity statistics of raw species read counts. (a) histogram of total taxonomic species abundance (read) counts for each of the metagenomic soil samples **(b)** species accumulation curve **(c)** plot to show, for each sample, the number of observed species before (*x*-axis) and after rarefication **(d)** rarefaction curve.

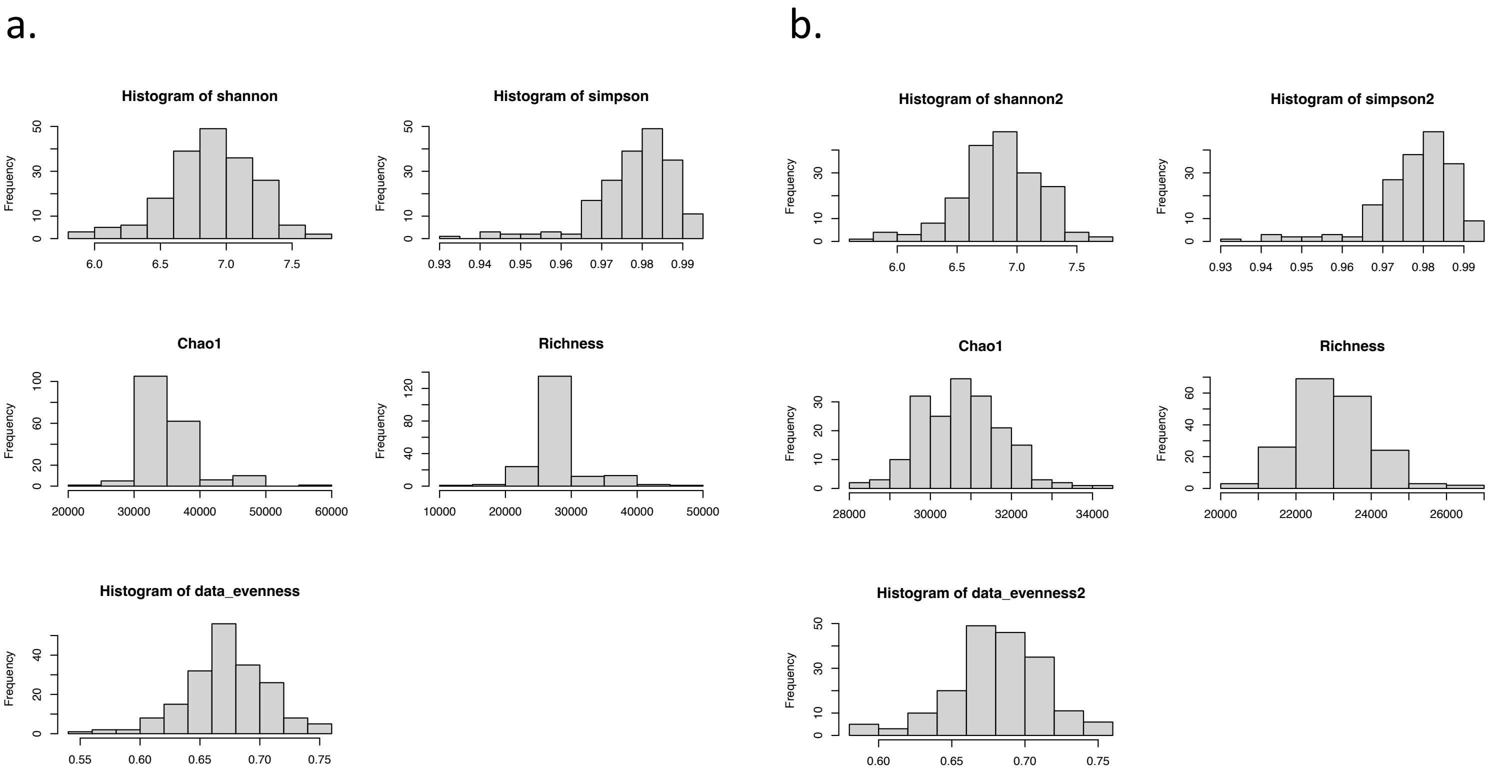

Supplementary Figure 18. Comparing taxonomic species diversity statistics pre and post-rarefication of read counts. Showing histograms of the range of alpha diversity values calculated for taxonomic species abundances across the metagenomic soil samples **(a)** for raw counts and **(b)** for rarefied counts for the following metrics; shannon index, simpson index, Chao1, species richness and Pielou's evenness.

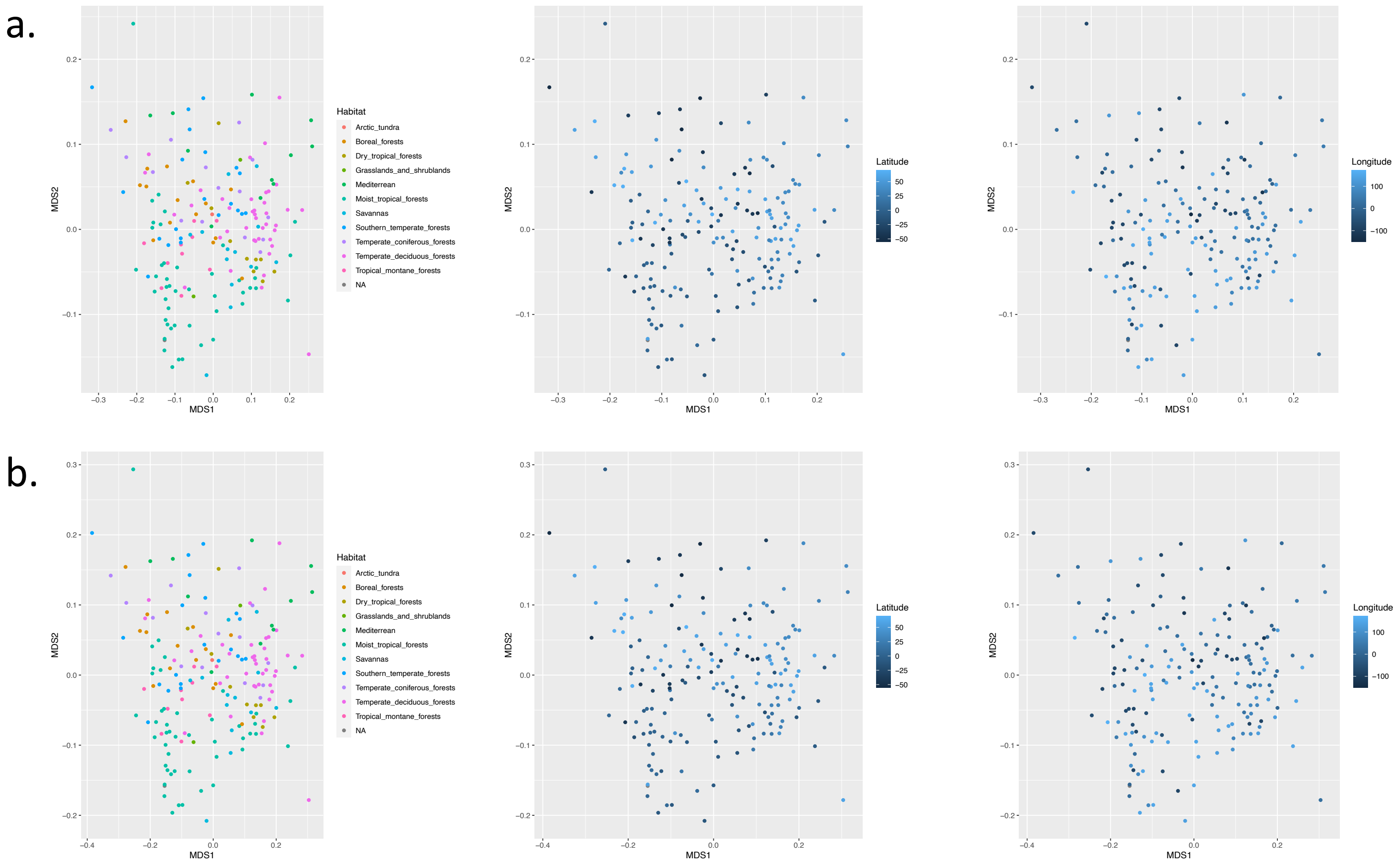

Supplementary Figure 19. Non-metric Multidimensional Scaling (NMDS) plots of beta diversity metrics. NMDS was used to represent the pairwise dissimilarity between metagenomic soil samples using **(a)** Bray-Curtis dissimilarity index and **(b)** Jaccard distance, in a 2-dimensional space denoted by MDS1 and MDS2. Scatter plots from left to right represent the colour coding of samples based on their habitat of origin, latitude and longitude respectively.

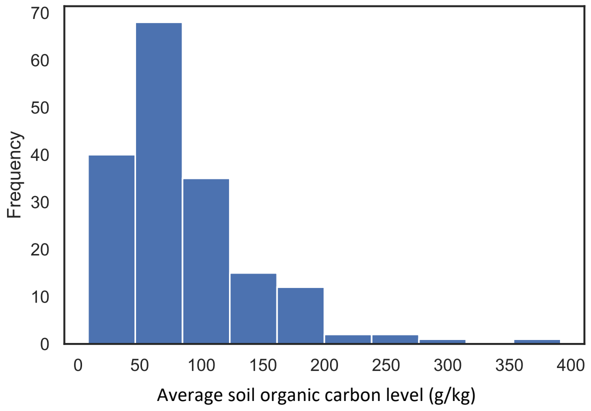

Supplementary Figure 20. Histogram showing the frequency distribution of average soil organic carbon (g kg^{-1}) levels for each location that the metagenomic samples were taken from. Soil organic carbon measurements and predictions are derived from SoilGrids and correspond to depth 0-5cm to align with the soil depth sampled for microbiome profiling.

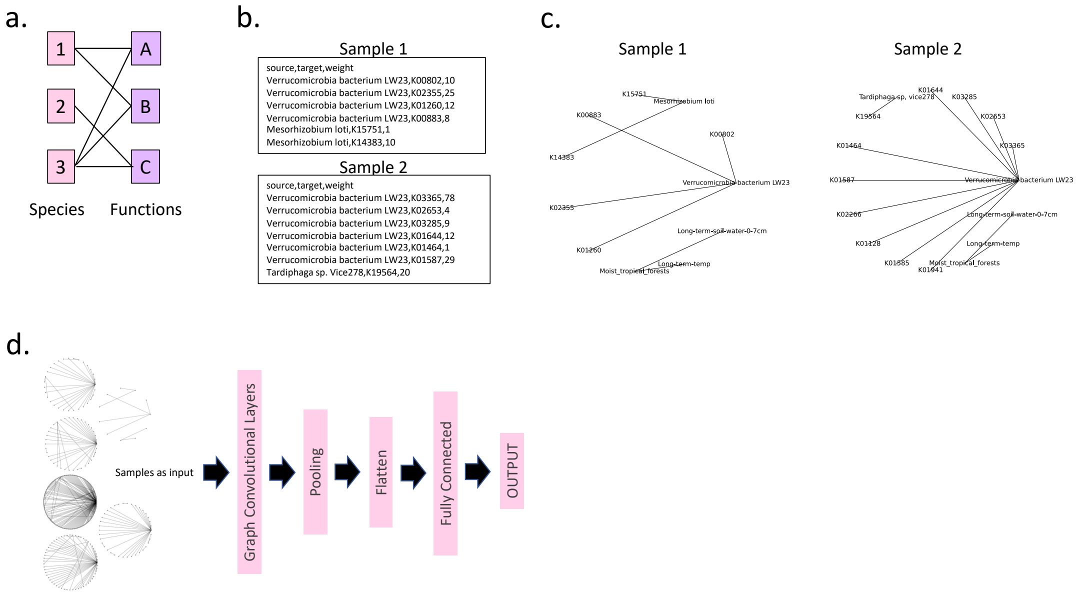

Supplementary Figure 21. Graph-based learning. (a) Depicts the theory behind how our per-sample graphs are constructed **(b)** Example of the format of edge lists for two samples **(c)** Showing sample graphs for two samples ("Small-graph" examples) **(d)** architecture of our DGCNN to predict SOC from microbial species, functions and environmental features.

Supplementary Tables

Supplementary Table 1. Output from the metagenomic bioinformatic workflow that was developed for this study. Including the proportion of reads passing Quality Control or QC (trimming and de-duplication) and subsequently the proportion of these reads that were aligned/classified by the different software's. These figures provide an average across the global soil metagenomic samples under analysis.

Software	% Reads after QC	Aligned/ classified QC reads (%)	% QC Read pairs with species assigned	% QC Read pairs with functional annotation
Kraken2/Bracken	93.0	90.3	29.4	-
Diamond/Megan	93.0	84.1	57.1	34.5
Humann3 ref50	93.0	52.3	-	10.1

Supplementary Table 2. Results of statistical tests for comparison of alpha diversity statistics. To assess the impact of the 11 represented habitats on the diversity of the microbiome, we used the analysis of variance (ANOVA) test and we show; Degrees of Freedom (DF), sum of squares (Sum Sq), mean squares (Mean Sq), *F-statistic* (*F value*) and *F-distribution* similar to *p-value* (Pr(>*F*)). For continuous variables Longitude and Latitude we used general linear models (GLMs), we also used GLMs to asses interaction between Habitat, Latitude and Longitude and the alpha diversity of the microbiome. For GLMs we show *t-values* and associated *p* (Pr(>|*t*|)). We only report results significant at *p*<0.05.

	**TEST: ANOVA**	**INDEPENDENT VARIABLE: Habitat**			
**Metric**	**DF**	**Sum Sq**	**Mean Sq**	***F* value**	***Pr(>F)***
**Shannon**	10	5.919	0.5919	7.353	1.14e-09
**Simpson**	10	0.005	0.0005	7.249	1.59e-09
**Evenness**	10	0.070	0.007	8.899	9.79e-12
**Species Richness**	10	7.488e+08	74876961	6.057	7.15e-08
**Chao1**	10	7.315e+08	73150859	6.212	4.33e-08

	**TEST: GLM (three-factor ANOVA)**	**INDEPENDENT VARIABLE: Habitat + Latitude + Longitude**			
**Metric**	**Variable**	**Estimate**	**Std. Error**	***t* value**	***Pr*(>|*t*|)**
**Shannon**	Latitude<br>Longitude	-0.0034136<br>-0.0007948	0.0014473<br>0.0002705	-2.359<br>-2.938	0.01947<br>0.00376
**Simpson**	Latitude<br>Longitude	-1.221e-04<br>-2.504e-05	4.317e-05<br>8.069e-06	-2.828<br>-3.103	0.00525<br>0.00224
**Evenness**	LatitudeLongitude	-3.296e-04<br>-1.070e-04	1.402e-04<br>2.621e-05	-2.351<br>-4.082	0.0199<br>6.84e-05
**Species Richness**	Longitude	14.954	3.262	4.584	8.76e-06
**Chao1**	Longitude	14.201	3.198	4.441	1.6e-05

	**TEST: GLM**	**INTERACTING VARIABLES: Habitat * Latitude * Longitude**	
**Metric**	**Interacting Variables**	***t* value**	***Pr*(>|*t*|)**
**Shannon**	Habitat-Moist_tropical_forests*Longitude<br>Habitat-Temperate_deciduous_forests*Longitude	-2.825<br>2.140	0.005387<br>0.034064
**Simpson**	Habitat-Moist_tropical_forests*Latitude<br>Habitat-Moist_tropical_forests*Longitude<br>Habitat-Moist_tropical_forests*Latitude*Longitude	-2.141<br>-2.488<br>2.040	0.0340<br>0.0140<br>0.0432
**Evenness**	Habitat-Moist_tropical_forests*Latitude<br>Habitat-Moist_tropical_forests*Longitude<br>Habitat-Temperate_deciduous_forests*Longitude<br>Habitat-Moist_tropical_forests:Latitude*Longitude<br>Habitat-Temperate_deciduous_forests*Latitude*Longitude	-2.551<br>-2.612<br>2.264<br>2.154<br>-2.088	0.01176<br>0.00995<br>0.02503<br>0.03286<br>0.03859
**Species Richness**	Habitat-Moist_tropical_forests*Latitude<br>Habitat-Southern_temperate_forests*Longitude	2.755<br>-2.166	0.00663<br>0.03194
**Chao1**	Habitat-Moist_tropical_forests*Latitude	3.143	0.00203

Supplementary Table 3. Results of statistical tests for comparison of beta diversity statistics. To assess the impact of the 11 represented habitats, Longitude and Latitude (and their potential interaction) on the (Bray Curtis and weighted UniFrac) beta diversity of the microbiome, we used the PERMANOVA test and we show; Degrees of Freedom (DF), sum of squares (Sum Sq), R2, F-statistic (*F* value) and F-distribution similar to *p*-value (Pr(>F)).

**Bray Curtis**	**DF**	**Sum Sq**	**Mean Sq**	***F* value**	***Pr*(>*F*)**
**Habitat**	10	2.9058	0.26206	7.2796	0.0001
**Latitude**	1	0.1321	0.01191	3.3087	0.0080
**Longitude**	1	0.2316	0.02089	5.8027	0.0001
**Habitat*Latitude**	9	0.7409	0.06682	2.0623	0.0002
**Habitat*Longitude**	8	0.7306	0.06589	2.2878	0.0001
**Latitude*Longitude**	1	0.0669	0.00604	1.6772	0.1037
**Habitat*Latitude*Longitude**	8	0.4923	0.04440	1.5416	0.0091
**UniFrac (weighted)**	**DF**	**Sum Sq**	**Mean Sq**	***F* value**	***Pr*(>*F*)**
**Habitat**	10	0.9603	0.27785	7.9395	0.0001
**Latitude**	1	0.0388	0.01123	3.2087	0.0134
**Longitude**	1	0.0860	0.02487	7.1065	0.0001
**Habitat*Latitude**	9	0.2172	0.06284	1.9951	0.0009
**Habitat*Longitude**	8	0.2277	0.06589	2.3536	0.0001
**Latitude*Longitude**	1	0.0220	0.00636	1.8160	0.1143
**Habitat*Latitude*Longitude**	8	0.1504	0.04352	1.5543	0.0252

Supplementary Table 4. Comparing ML models for prediction of soil organic carbon using alpha diversity. Showing ML model performance (using best parameters after fine tuning) for a range of metrics including Mean Absolute Error (MAE) on the training dataset (Train MAE), MAE on the held-out test set (Test MAE), the mean and standard deviation of the MAE value after 5-fold cross-validation (Mean MAE CV, SD MAE CV), R2 values for the training and held-out test dataset (Train r2 and Test r2) and the explained variance for the training and held-out test dataset. In the upper part of the table, the alpha diversity metrics used for model training include species richness, Chao1, Pielou's evenness, Shannon index and Simpson index. In the lower part of the table the Hill numbers are used for model training i.e., q is equal to 0, 1 and 2.

Estimator	Logistic Regression	Random Forest	SVM	KNN	Xgboost	LightGBM	Gradient Boosting	GP Regression
**Test r^2**	0.131	-0.031	-0.062	0.102	-0.071	-0.005	-0.11	0.149
**Test MAE**	35.138	36.303	36.395	35.369	37.548	38.452	38.777	34.251
**Test explained variance**	0.137	0.048	0.039	0.104	0.04	0	0	0.155
**Train r^2**	0.175	0.087	0.067	1	0.012	0	-0.049	0.228
**Train MAE**	38.569	37.488	38.173	0	38.885	42.769	40.739	36.31
**Train explained variance**	0.175	0.126	0.121	1	0.067	0	0	0.228
**Mean MAE CV**	41.251	41.026	39.3801	42.615	40.926	42.916	41.172	40.817
**SD MAE CV**	4.703	4.384	3.489	5.285	3.574	6.201	4.321	3.373
**Estimator**	**Logistic Regression**	**Random Forest**	**SVM**	**KNN**	**Xgboost**	**LightGBM**	**Gradient Boosting**	**GP Regression**
**Test r^2**	-0.042	-0.031	-0.062	0.102	-0.071	-0.005	-0.11	0.149
**Test MAE**	40.156	36.303	36.395	35.369	37.548	38.452	38.777	34.251
**Test explained variance**	-0.041	0.048	0.039	0.104	0.04	0	0	0.155
**Train r^2**	0.074	0.087	0.067	1	0.012	0	-0.049	0.228
**Train MAE**	41.636	37.488	38.173	0	38.885	42.769	40.739	36.31
**Train explained variance**	0.074	0.126	0.121	1	0.067	0	0	0.228
**Mean MAE CV**	43.661	41.026	39.380	42.615	40.926	42.916	41.172	40.817
**SD MAE CV**	5.430	4.384	3.489	5.285	3.574	6.201	4.321	3.373

Supplementary Table 5. Environmental variables collected to match our metagenomic soil samples based on geo-location.

**Soil Temperature 0 – 7cm (day of sample)**	soiltemp0to7cm	Temperature of the soil (0 - 7 cm) of the ECMWF Integrated Forecasting System. The surface is 0 cm. Soil temperature is set at the middle of each layer (degrees Celsius)
**Soil Temperature Difference to Average**	soiltemp_0_7_diff	Take 20-year average for an area and measure how much it deviates in the year in which sampling of the microbiome was conducted to give an indication of whether it was a normal year or particularly warm/cold (degrees Celsius)
**Soil Water 0 – 7cm (day of sample)**	soilwater0to7cm	Volume of water in soil layer 1 (0 - 7 cm) of the ECMWF Integrated Forecasting System. The surface is at 0 cm. Volumetric soil water is associated with soil texture (or classification), soil depth, and the underlying groundwater level (m^{-3} m^{-3})
**Soil Water 0 – 7cm Difference to Average**	soilwater_0_7_diff	Take 20-year average for an area and measure how much it deviates in the year in which sampling of the microbiome was conducted to give an indication of whether it was a normal year or particularly dry/wet (m^{-3} m^{-3})
**Total precipitation (day of sample)**	totalrain	Total rainfall for the specific day the sample was taken (m)
**Total precipitation Difference to Average**	totalrain_diff	Take 20-year average for an area and measure how much it deviates in the year in which sampling of the microbiome was conducted to give an indication of whether it was a normal year or particularly dry/wet (m)
**Temperature long term**	temp_long	Take 20-year average air temperature for a locale
**Temperature (day of sample)**	temp	Temperature on the day the microbiome sample was taken (at lunchtime) (degrees Celsius)
**Total temperature Difference to Average**	temp_diff	Take 20-year average for an area and measure how much it deviates in the year in which sampling of the microbiome was conducted to give an indication of whether it was a normal year or particularly hot/cold (degrees Celsius)
**Type of low vegetation**	type_low_veg	Static value-options detailed in the main text as to the ten types of vegetation that the ECMWF model considers as low.
**Type of high vegetation**	type_high_veg	Static value-options detailed in the main text as to the ten types of vegetation that the ECMWF model considers as high.
**Coarse fragments**	coarse_depth	Volumetric fraction of coarse fragments (cm^{-3} $10cm^{-3}$ (vol%))
**Soil Organic Carbon density**	orgcarbon_depth	Volumetric fraction of soil organic carbon across a range of depths ($g\ kg^{-1}$)
**Sand**	Sand_depth	Volumetric fraction of sand-proportion of sand particles in fine earth fraction ($g\ 100g^{-1}$ (%))
**Clay**	Clay_depth	Volumetric fraction of clay-proportion of clay particles in fine earth fraction ($g\ 100g^{-1}$ (%))
**Silt**	Silt_depth	Volumetric fraction of silt-proportion of silt particles in fine earth fraction ($g\ 100g^{-1}$ (%))
**Nitrogen**	Nitro_depth	Total Nitrogen ($g\ kg^{-1}$)
**pH**	pH_depth	Soil pH
**Bulk Density**	bulk_density	Bulk density of the fine earth fraction ($kg\ dm^{-3}$)
**Cation Exchange Capacity**	Cation_depth	Cation exchange capacity of the soil (cmol(c) kg^{-1})
**Most probable soil class**	Soil_class	Most probable class of the soil. Static value

Supplementary Table 6. Comparing ML models for prediction of soil organic carbon for combined environmental, taxonomic and functional abundances plus alpha diversity metrics-22 features after feature selection. Showing ML model performance (using best parameters after fine tuning) for a range of metrics including Mean Absolute Error (MAE) on the training dataset (Train MAE), MAE on the held-out test set (Test MAE), the mean and standard deviation of the MAE value after 5-fold cross-validation (Mean MAE CV, SD MAE CV), Root mean Squared Error (RMSE) on the training dataset (Train RMSE) and test dataset (Test RMSE), R2 values for the training and held-out test dataset (Train r2 and Test r2) and the explained variance for the training and held-out test dataset.

Estimator	Logistic Regression	Random Forest	SVM	KNN	Xgboost	LightGBM	Gradient Boosting	GP Regression
**Test r^2**	0.62	0.62	0.52	0.70	0.53	0.25	0.02	0.57
**Test MAE**	20.72	23.53	24.60	20.83	25.17	28.07	37.46	23.54
**Test RMSE**	29.38	27.53	32.96	26.28	32.67	41.35	47.99	31.27
**Test explained variance**	0.63	0.65	0.54	0.70	0.53	0.25	0	0.59
**Train r^2**	0.70	0.54	0.35	1	0.72	1	0.01	0.94
**Train MAE**	22.33	23.39	29.87	0	11.20	0	43.26	10.65
**Train RMSE**	32.65	34.92	48.07	0	5.64	0	59.71	14.81
**Train explained variance**	0.70	0.55	0.38	1	0.73	1	0	0.94
**Mean MAE CV**	62.11	29.20	32.12	28.90	29.29	34.62	43.43	35.88
**SD MAE CV**	66.17	5.70	8.88	8.30	5.66	5.28	10.70	9.23

Supplementary Table 7. Outlining the output files/tables from the metagenomic bioinformatic workflow that was developed for this study. The workflow was initially aimed as quantification of species level abundances as such only species level information is outlined here. The term ReadID denotes the name for a sequencing read as derived from its fastq file.

Classification/ Alignment Software	Purpose	Output Information	Output File Format (TAB separated)
Kraken2	Taxonomy	Species assigned to each read	ReadID<br>Taxonomy-ID (NCBI Taxonomy identifier number)<br>Species-Name
Kraken2	Taxonomy	Per species abundance counts (derived via Bracken)	Species-Name<br>Taxonomy-ID (NCBI Taxonomy identifier number)<br>Taxonomy_level (S denoted species level)<br>kraken_assigned_reads (kraken2 count of classified reads)<br>added_reads (count of Bracken identified reads to add to Kraken2's)<br>new_est_reads (Total count of assigned reads Bracken+kraken2)<br>fraction_total_reads (new_est_reads as a fraction of all reads)
Diamond/MEGAN	Taxonomy	Species assigned to each read	ReadID<br>Taxonomy-ID (NCBI Taxonomy identifier number)<br>Species-Name
Diamond/MEGAN	Taxonomy	Per species abundance counts	Species-Name<br>Taxonomy-ID (NCBI Taxonomy identifier number)<br>Taxonomy_level (S denoted species level)<br>diamond_assigned_reads (Diamond count of aligned reads)
Diamond/MEGAN	Function	Functions assigned to each read	ReadID<br>KEGG-ID "\s" KEGG-ID Description

Diamond/MEGAN	Function	Per function abundance counts	KEGG-ID "\s" KEGG-ID Description<br>Read count per KEGG-ID
Humann3	Function	Functions assigned to each read	ReadID<br>KEGG-ID<br>RefSeq contig KEGG ID came from (UniRef50)
Humann3	Function	Per function abundance counts	KEGG-ID<br>Read count per KEGG-ID

Supplementary Table 8. Summarising the information gained as output from the metagenomic bioinformatic workflow that was developed for this study.

**Output**	**Kraken2/ Bracken**	**DIAMOND/ MEGAN**	**Humann3**
Taxonomic Species Abundance	✓	✓	✗
KEGG Orthologue Abundances	✗	✓	✓
Quality/Certainty Scores Available	✓	✓	✓
Metrics for Read Mapping (e.g., can we calculate the length of the read that was aligned)	✓	✓	✓

Supplementary Table 9. Comparing taxonomic species diversity statistics pre- and post-rarefication of read counts. Showing Pearson correlation coefficients (R) of the range of alpha diversity values calculated for taxonomic species abundances across the metagenomic soil samples.

Comparison	Richness (Pre)	Chao1 (Pre)	Shannon (Pre)	Simpson (Pre)	Evenness (Pre)
Richness (Post)	0.248				
Chao1 (Post)		0.261			
Shannon (Post)			0.999		
Simpson (Post)				0.999	
Evenness (Post)					0.982

Supplementary Table 10. Parameter training during hyperparameter optimization for comparison of commonly used deterministic ML methods. Showing the parameters that were tuned using Grid Search and the range of trialled hyperparameters.

**Regressor**	**Hyperparameter tuning**
LOGISTIC REGRESSION	fit_intercept:[True, False], normalize:[True, False], copy_X:[True, False]
RANDOM FOREST	criterion: ['mse', 'mae'], min_samples_leaf: [1, 2, 3, 4, 5, 6, 7, 8, 9, 10]<br>max_depth: [1, 2, 3, 4, 5, 6, 7, 8, 9, 10], min_samples_split: [2, 5, 10]
SVM	kernel: ['linear', 'poly', 'rbf'], degree: [0, 1, 2, 3, 4, 5, 6], coef0: scipy.stats.expon(scale=1)<br>max_iter: [int(x) for x in np.linspace(start=100, stop=4000, num=10)]<br>gamma: scipy.stats.expon(scale=.1), C: [1, 2, 3, 4, 5, 6, 7, 8, 9, 10]
KNN	n_neighbors: [1, 2, 3, 4, 5, 6, 7, 8, 9, 10, 11, 12, 13, 14, 15, 16, 17, 18, 19, 20]<br>leaf_size: [1, 2, 3, 4, 5], weights:['uniform', 'distance']<br>algorithm:['auto', 'ball_tree','kd_tree','brute']
XGBOOST	max_depth: [2, 3, 4, 5, 6, 7, 8, 9, 10, 15, 20, 25], subsample: [0.2, 0.5, 0.6, 0.7, 0.8, 0.9, 1]<br>learning_rate: scipy.stats.expon(scale=1), min_child_weight: scipy.stats.expon(scale=10)<br>max_delta_step: [0, 1, 2], objective:['reg:linear'], gamma: scipy.stats.expon(scale=20)<br>n_estimators: [50, 150, 200, 250, 300, 350, 400, 450, 500]
GRADIENT BOOSTING	criterion: ['mse', 'mae', 'friedman_mse'], min_samples_leaf: [50, 100, 200],<br>max_depth: [3, 5, 6, 7, 8, 10], min_samples_split: [100, 200, 300, 400, 500],<br>max_features: ['sqrt'], learning_rate: [0.05, 0.1, 0.2],<br>n_estimators: [20, 40, 60, 80], 'clf__subsample': [0.2, 0.5, 0.6, 0.7, 0.8, 0.9, 1]
LIGHT GBM	num_leaves: [10, 20, 50, 100, 200], subsample: [0.2, 0.5, 0.6, 0.7, 0.8, 0.9, 1]<br>min_data_in_leaf: [10, 25, 50, 75, 100], max_depth: [3, 5, 6, 7, 8, 10, 15, 20, 25]<br>learning_rate: scipy.stats.expon(scale=1)<br>n_estimators: [50, 150, 200, 250, 300, 350, 400, 450, 500]
GAUSSIAN PROCESS	normalize_y:[True,False], copy_X_train:[True, False],<br>alpha: [1e-2, 1e-4, 1e-6, 1e-8, 1e-10, 1e-12] , n_restarts_optimizer: [10, 20, 30]

Supplementary Table 11. Selected best performing classic ML models and their hyperparameters. During comparison of commonly used deterministic ML methods, this table shows the parameters that were selected after tuning using Grid Search and the range of trialled hyperparameters from Supplementary Table 10.

FEATURE SET	REGRESSOR	hyperparameters
22 selected microbial + environmental features	SVM<br><br>Epsilon-Support Vector Regression	Pipeline(steps=[('scl', MinMaxScaler()), ('clf', svm.SVR(C=12, gamma=0.09744012947089305, max_iter=100))])

Supplementary References

1. Gardiner LJ, Haiminen N, Utro F, Parida L, Seabolt E, Krishna R, et al. Re-purposing software for functional characterization of the microbiome. Microbiome. 2021 Dec;9(1):4.

2. Zhang B, Zeng F, Gao X, Shareef M, Zhang Z, Yu Q, et al. Groundwater depth alters soil nutrient concentrations in different environments in an arid desert. Front Environ Sci. 2022 Aug 22;10:939382.

3. Bahram M, Hildebrand F, Forslund SK, Anderson JL, Soudzilovskaia NA, Bodegom PM, et al. Structure and function of the global topsoil microbiome. Nature. 2018 Aug;560(7717):233–7.
